# Anatomical Mapping of the Facial Artery and Facial Vein Relative to Bony Landmarks: A Morphometric and Morphological Study for Navigating Vascular Clinical Risks

**DOI:** 10.1007/s00266-026-05934-9

**Published:** 2026-05-28

**Authors:** Rukiye Soyal, Betül Digilli Ayaş, Aynur Emine Çiçekcibaşı, Süleyman Bakdık

**Affiliations:** 1 Department of Anatomy, Faculty of Medicine, Necmettin Erbakan University, 42090 Meram, Konya Turkey; 2 Department of Radiology, Faculty of Medicine, Necmettin Erbakan University, 42090 Meram, Konya Turkey

**Keywords:** Bony landmarks, Computed tomography angiography, Facial artery, Facial vein, Morphology, Morphometry

## Abstract

**Background:**

Facial vascular anatomy exhibits significant complexity and individual diversity, which are central to managing potential procedural complications. This study aims to provide a comprehensive topographic map and morphometric analysis of the facial artery (FA) and facial vein (FV) relative to stable bony landmarks to characterize their distribution and morphological variations.

**Methods:**

Head and neck computed tomography angiography images of 200 patients (400 FA and FV) were analyzed. The detailed courses and variations in the FA and FV were evaluated, and their distances to various bony landmarks and anatomical planes were measured.

**Results:**

We identified 10 FA and 3 FV types, including one new FV variation. The most common FA type was the lateral nasal type, which predominantly coursed medial to the nasolabial fold. In the infraorbital region, areas outside 1.64 cm (right) and 1.55 cm (left) of the rhinion represent lower vascular density zones. For the medial nasolabial fold, areas closer than 1.53 cm (right) and 1.46 cm (left) from the anterior nasal spine superiorly, and nearer than 2.23 cm on the right and 2.07 cm on the left from the alveolar process of the maxilla inferiorly, represent lower vascular density zones relative to the FA.

**Conclusions:**

A detailed anatomical framework of the FA and FV was established, providing a stable reference for anatomical navigation and vascular clinical risk mitigation. The distance between vessels and bony landmarks was greater in males compared to females, and in younger individuals compared to older ones.

**Level of Evidence IV:**

This journal requires that authors assign a level of evidence to each article. For a full description of these Evidence-Based Medicine ratings, please refer to the Table of Contents or the online Instructions to Authors www.springer.com/00266.

## Introduction

In recent years, non-invasive cosmetic applications have become important in aesthetic medicine due to the lower risk of iatrogenic damage, short recovery period, and high patient satisfaction rates [1]. Among these, hyaluronic acid-based interventions are widely used for rejuvenation and volume restoration in the nose, forehead, cheek, and nasolabial fold (NLF) regions and have become a popular alternative to surgical rejuvenation of the face [2, 3]. However, such aesthetic procedures can cause serious vascular complications like erythema, tissue necrosis, blindness, paralysis, and rarely death, mostly from accidental vascular injury [1–4]. Therefore, a comprehensive understanding of the detailed anatomy, including the course, localization, and variations, of the facial artery (FA) and facial vein (FV), is critically important for minimizing the risk of iatrogenic injury and ensuring procedural safety during clinical interventions.

Recent studies have demonstrated that anatomical variations of FA and its branches are directly associated with the risk of vascular complications [1–5]. This situation has necessitated a more detailed identification of anatomical danger zones across different regions of the face. In this context, our study aims to perform a detailed morphometric and morphological analysis of various FA and FV anatomical features, including their branching patterns, courses, and variations. By establishing a topographic map relative to stable bony landmarks, we aim to define zones of lower vascular density to enhance clinical vigilance and anatomical awareness during various facial procedures.

## Materials and Methods

The local ethics committee approved the research with decision number 2024/5405.

### Patients

This retrospective study evaluated FA and FV on head and neck computed tomography angiography (CTA) images acquired between January 2020 and December 2024. Exclusion criteria were facial surgery, deformities affecting bone anatomy, vascular pathologies such as aneurysms, arteriovenous malformations, or vascular tumors, and significant artifacts on CTA images. Using G*Power 3.1.9.7 (Universität Düsseldorf, Düsseldorf, Germany) with an effect size of 0.5, *α*=0.05, and power=90%, the required sample size was 172 (86 per group). Of 472 CTA, 271 were excluded due to dental artifacts, unclear FA/FV branches, or age limits, leaving 201 bilaterally imaged patients. One patient had a double FA on the right and transverse FA on the left; only FA typing was done for this case. The final cohort comprised 200 participants (100 women, 100 men), equally distributed across four age groups (30–39, 40–49, 50–59, 60–69), selected to ensure adequate statistical power for age-related analyses.

### CTA Technique and Image Analyses

CTA scans were performed after contrast administration using a 64 slice-CT scanner (Siemens Somatom go.Up, Siemens Healthineers, Erlangen, Germany) with the following acquisition settings: 110 kV, 110 mA, 32 mm × 0.7 mm detector collimation, 1 mm slice thickness, and a 512 × 512 image matrix. Images were analyzed using 3D volume-rendered and multiplanar reconstructions on Syngovia workstations (Siemens, Germany). FA and FV distances to bone landmarks, FA branching, FA–NLF relationship, and FV course were bilaterally assessed, with additional vascular variations recorded. Measurements were done by an experienced radiologist and anatomist.

### Classification of Variations of the FA and FV

**Fig. 1:**
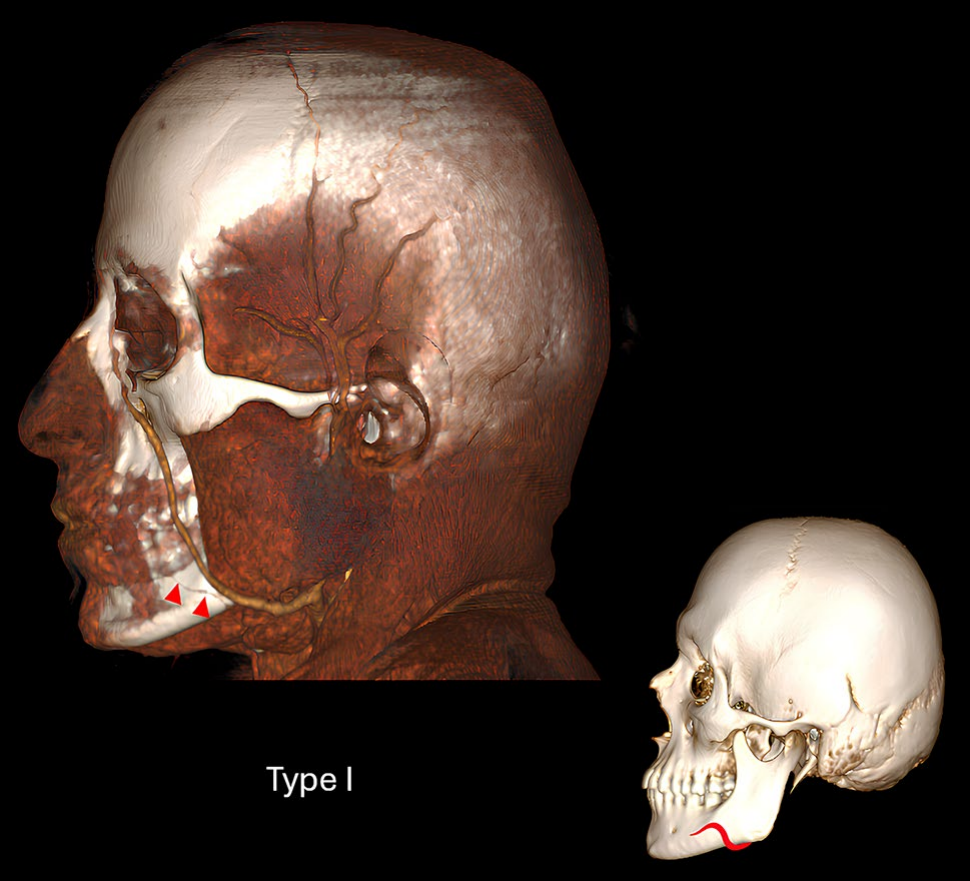
Three-dimensional volume rendering computed tomography angiography image and schematic illustration of the course type of Type I FA. FA is hypoplastic along the mandible and gives off no branches

**Fig. 2:**
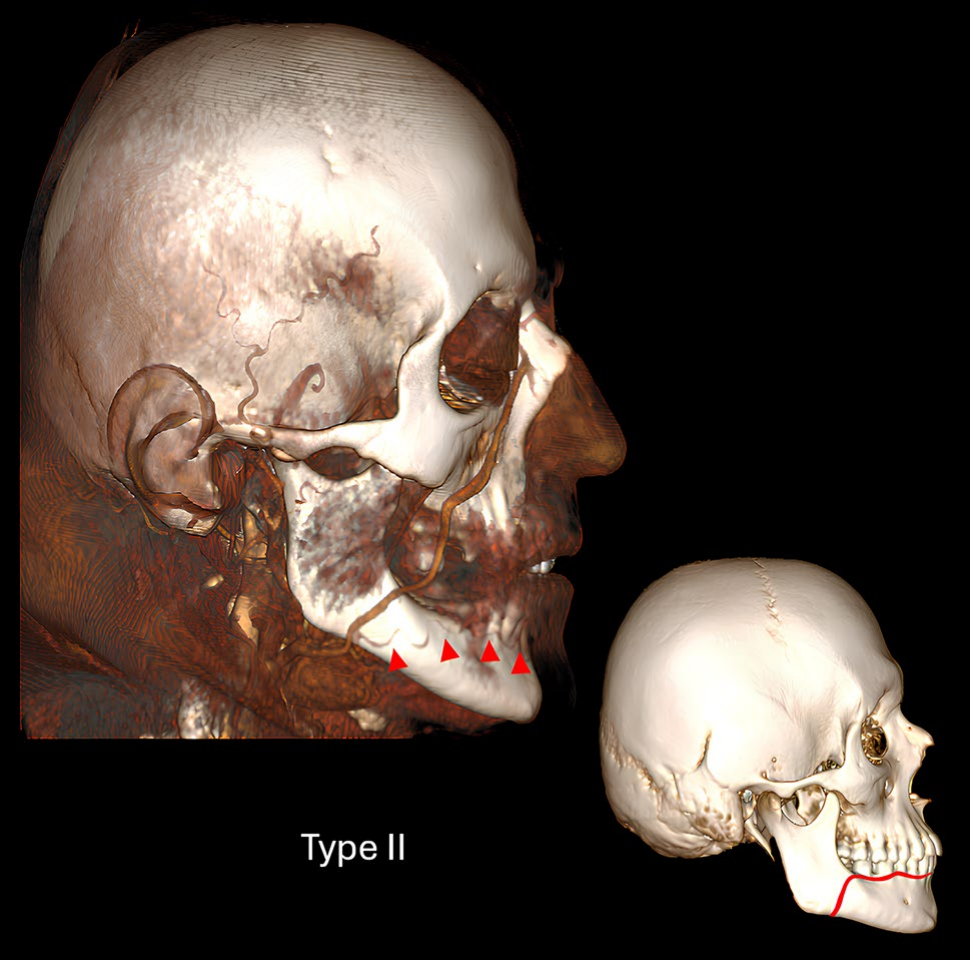
Three-dimensional volume rendering computed tomography angiography image and schematic illustration of the course type of Type II FA. FA terminates as the inferior labial artery

**Fig. 3:**
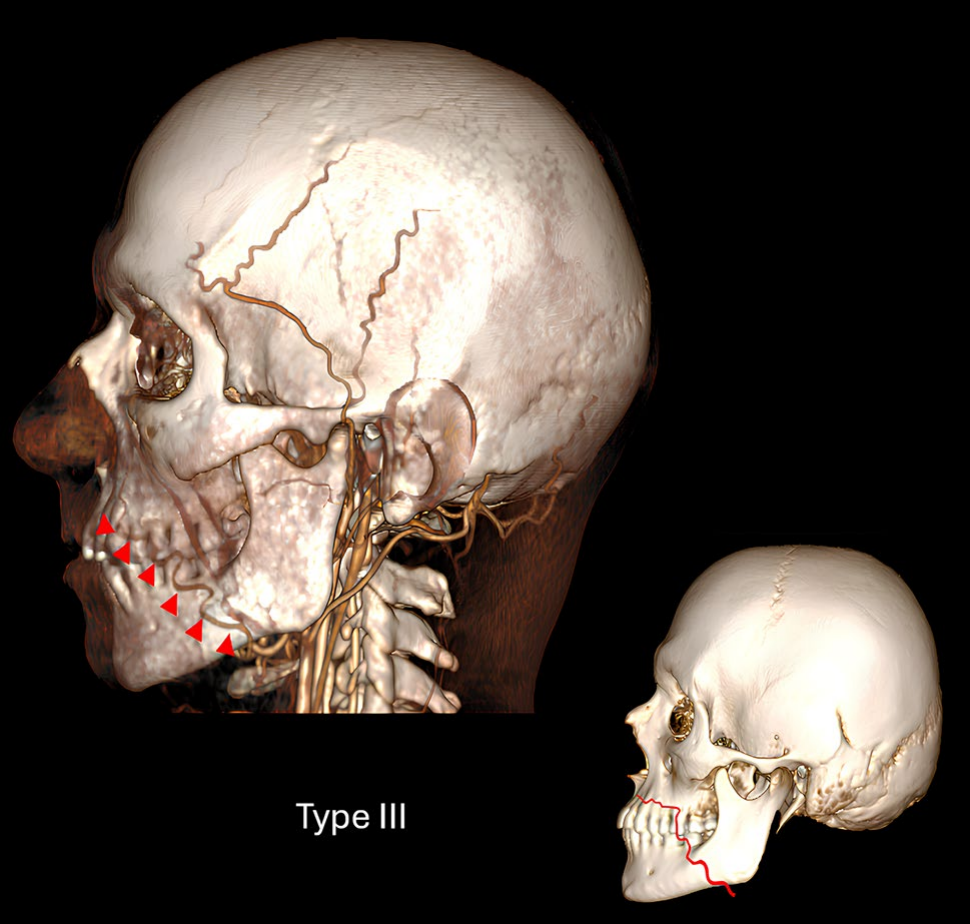
Three-dimensional volume rendering computed tomography angiography image and schematic illustration of the course type of Type III FA. FA terminates only as the superior labial artery without the inferior labial artery

**Fig. 4:**
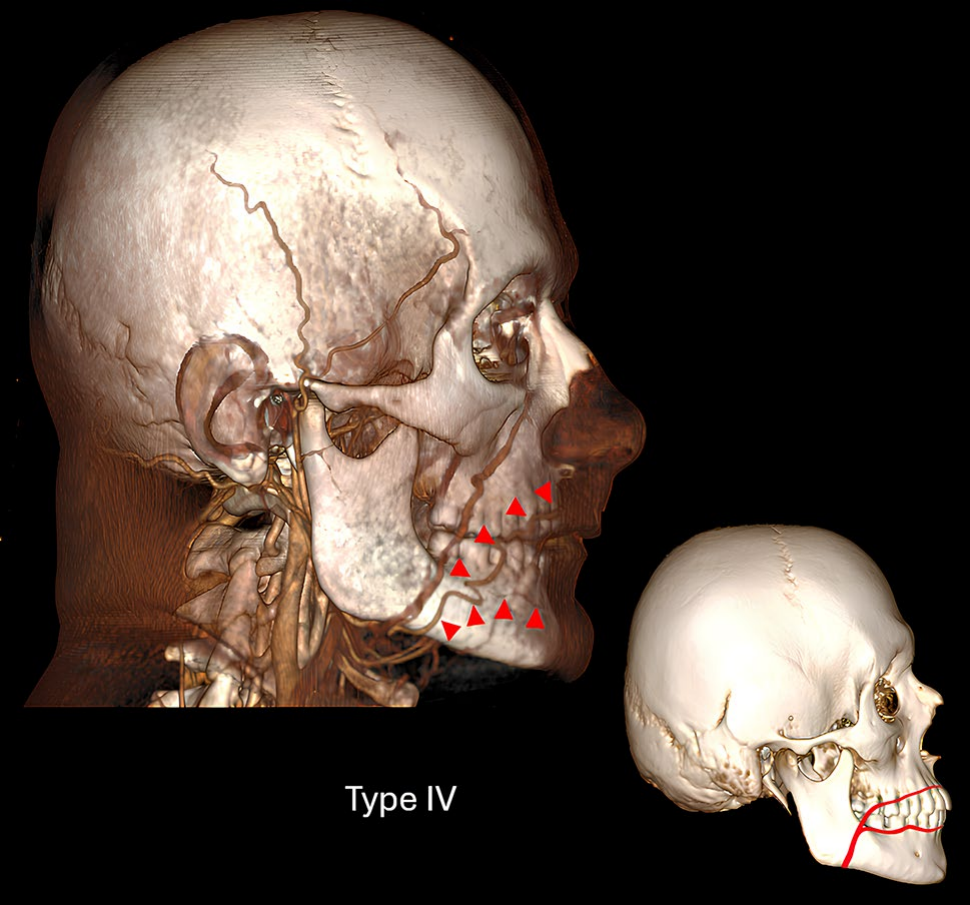
Three-dimensional volume rendering computed tomography angiography image and schematic illustration of the course type of Type IV FA. FA terminates as the superior labial artery

**Fig. 5:**
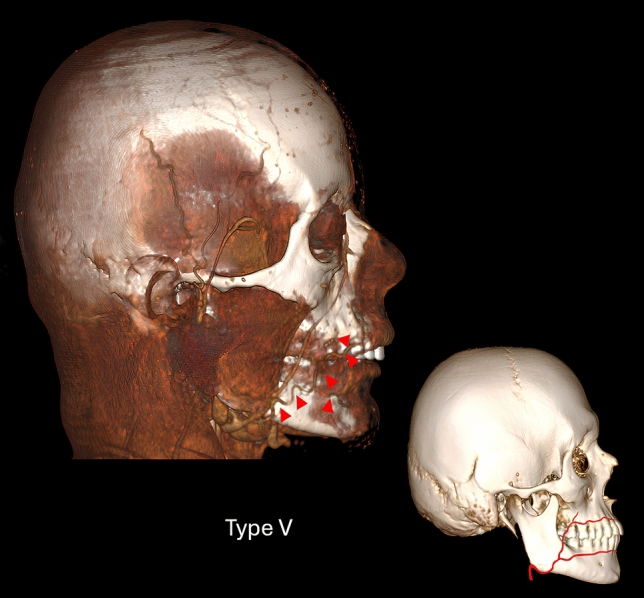
Three-dimensional volume rendering computed tomography angiography image and schematic illustration of the course type of Type V FA. FA terminates distal to the superior labial artery near nasolabial fold

**Fig. 6:**
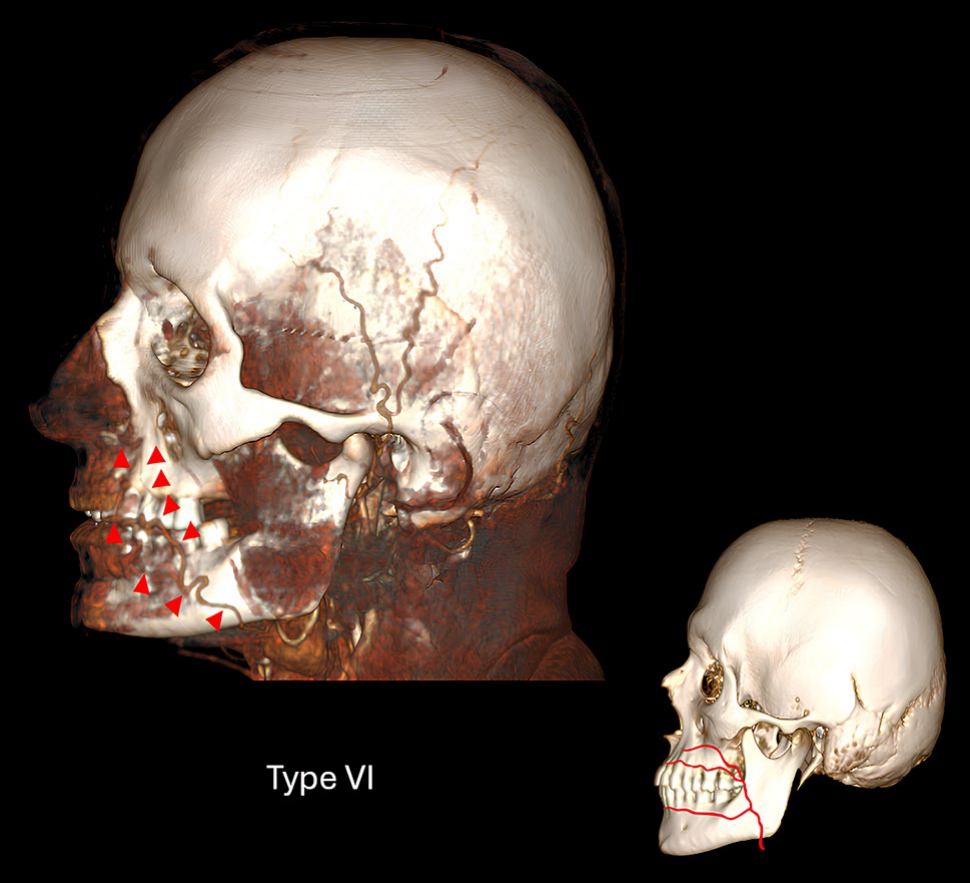
Three-dimensional volume rendering computed tomography angiography image and schematic illustration of the course type of Type VI FA. FA terminates as the inferior alar artery

**Fig. 7:**
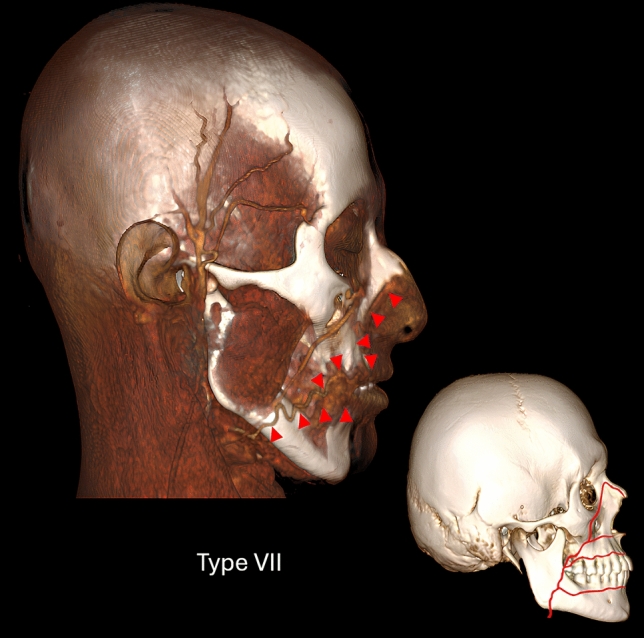
Three-dimensional volume rendering computed tomography angiography image and schematic illustration of the course type of Type VII FA. FA terminates as the lateral nasal artery

**Fig. 8:**
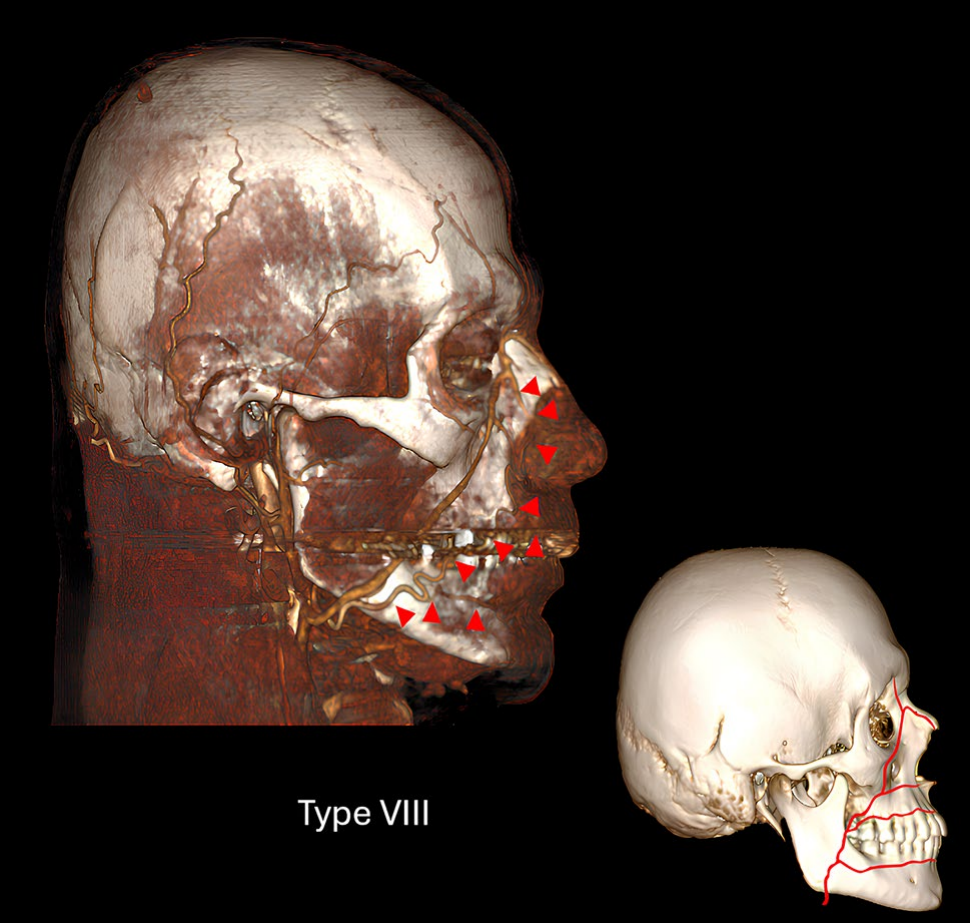
Three-dimensional volume rendering computed tomography angiography image and schematic illustration of the course type of Type VIII FA. FA terminates as the angular artery

**Fig. 9:**
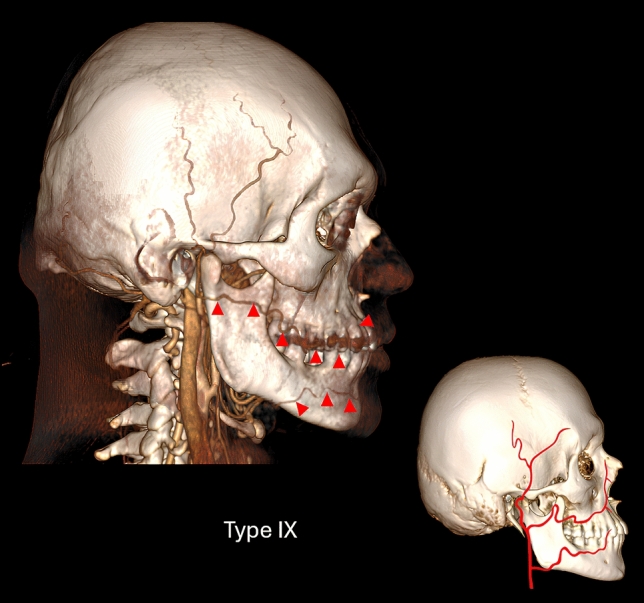
Three-dimensional volume rendering computed tomography angiography image and schematic illustration of the course type of Type IX FA. The type in which FA is hypoplastic and its remaining portion is supplied by a dominant transverse FA

**Fig. 10:**
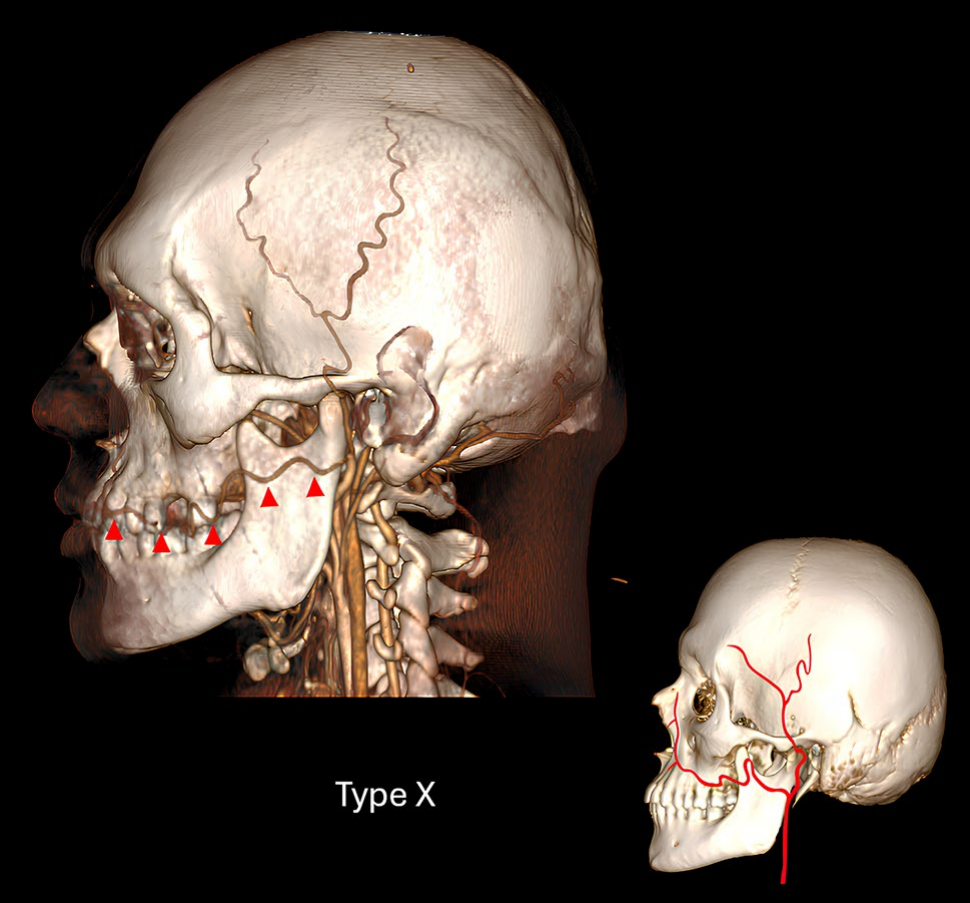
Three-dimensional volume rendering computed tomography angiography image and schematic illustration of the course type of Type X FA. FA arises from the external carotid artery at a higher level than normal and courses transversely on the face (transverse type)

**Fig. 11:**
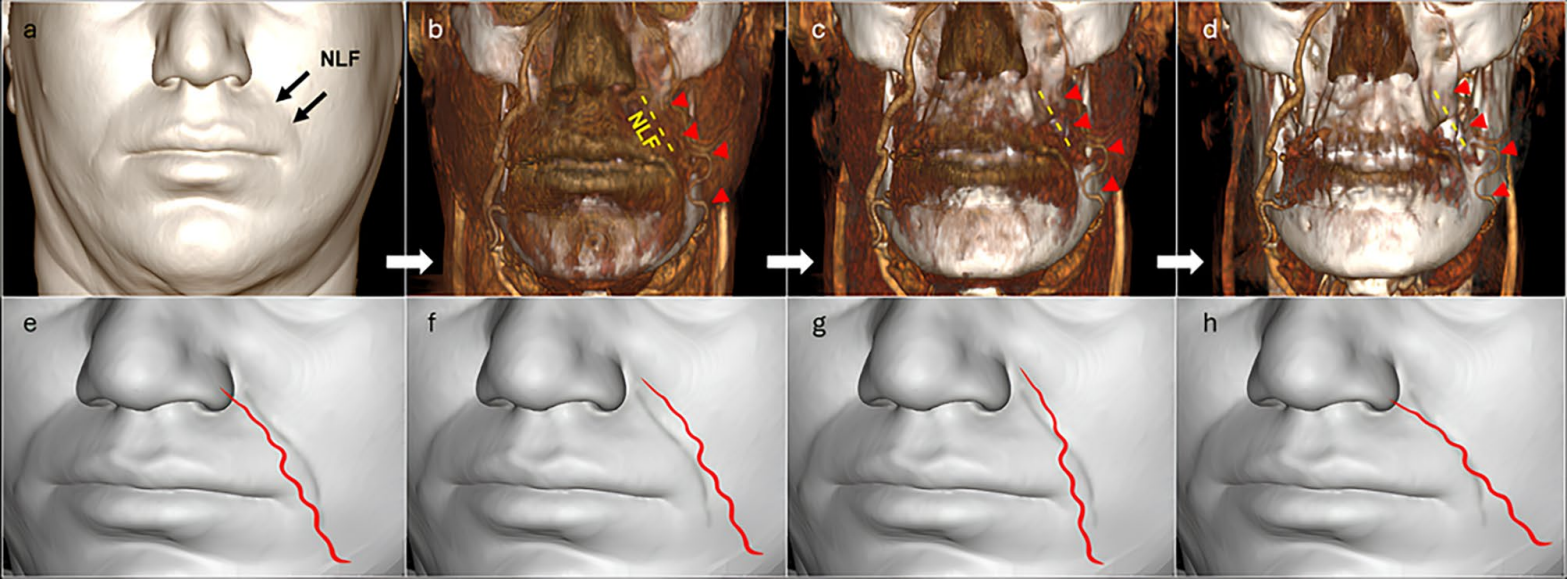
Location of the FA relative to the nasolabial fold. **a** Demonstration of the nasolabial fold in a 40-year-old woman's skin image obtained by three-dimensional volume rendering of computed tomography angiography. **b**–**d** In the image obtained by three-dimensional volume rendering of computed tomography angiography, the Type IX FA (red arrowhead) on the left side is located lateral to the nasolabial fold (yellow dashed line). Schematic illustrations of the FA located medial to the nasolabial fold (**e**), lateral to the nasolabial fold (**f**), crossing from medial to lateral (cross laterally) (**g**), and crossing from lateral to medial (crossing medially) (**h**). (NLF: Nasolabial fold)

**Fig. 12:**
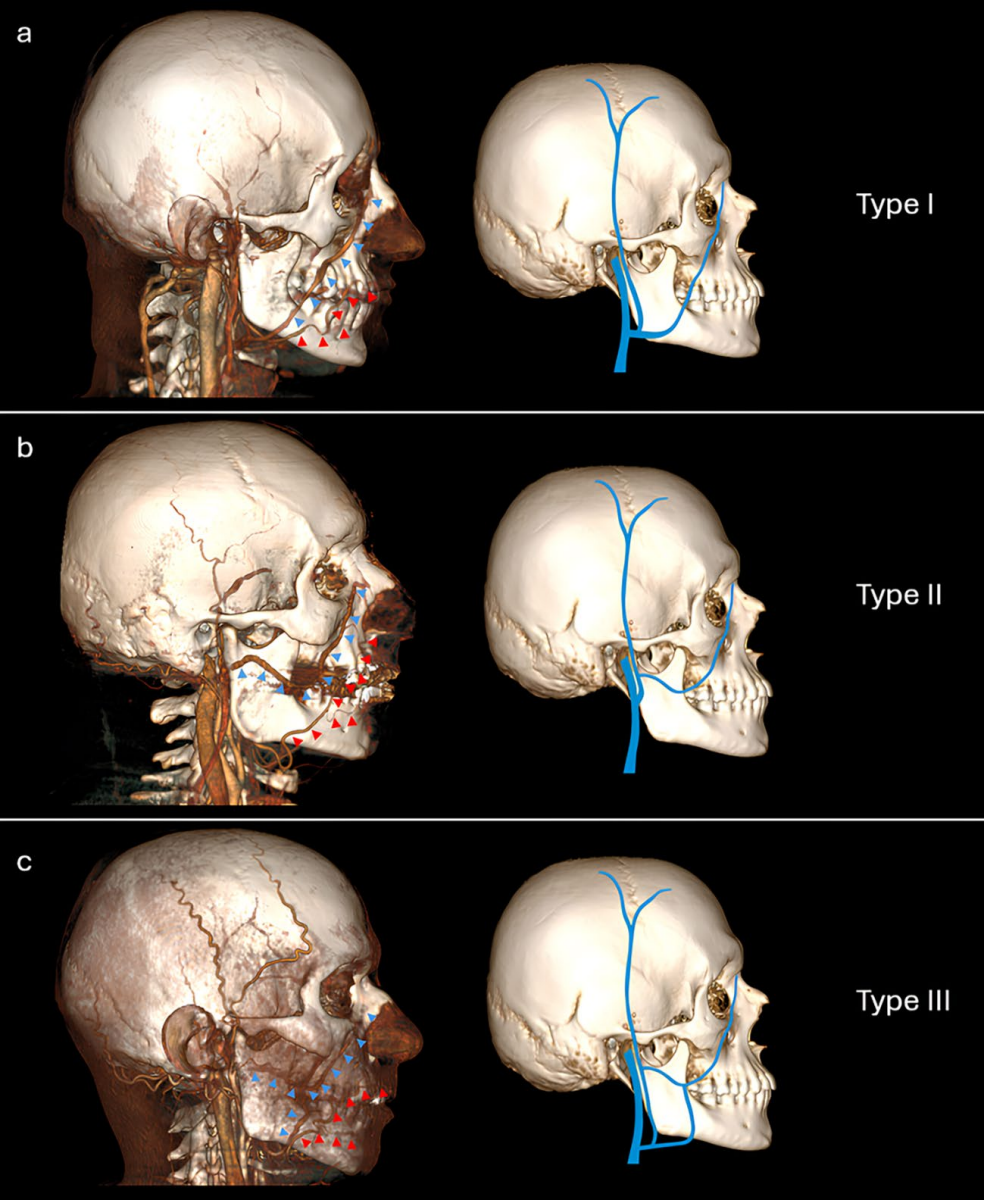
Three-dimensional volume rendering computed tomography angiography images and schematic illustrations of the course types of FV. **a** Type I: The FV passes through the mandible at the anterior margin of the masseter muscle and opens into the retromandibular vein. **b** Type II: The FV passes through the surface of the masseter muscle and opens into the superficial temporal vein.** c** Type III: The FV passes through the surface of the masseter muscle and anastomoses with a branch from the superficial temporal vein. (Blue arrowhead: FV, Red arrowhead: FA)

This study evaluated 14 different terminal branch types of the FA, previously described by various authors in the literature (Table 1, Figs. 1, 2, 3, 4, 5, 6, 7, 8, 9 and 10). [2, 4–9]. Additionally, the NLF was marked on the soft tissue between the nasal ala and the cheilion, and the FA position relative to the NLF (FA–NLF type) was classified by tracing from soft tissue to bone according to Wang et al.’s [3] methodology (Table 1 and Fig. 11). FV course types were also classified according to Wang et al. [10] (Table 1 and Fig. 12).

**Table 1:** Classification of types of the facial artery and facial vein

	Abbreviations
*FA Types*	
FA is hypoplastic on the mandible and gives off no branches (Fig. 1)	Type I
FA terminates as the inferior labial artery (Fig. 2)	Type II
FA terminates only as the superior labial artery without the inferior labial artery (Fig. 3)	Type III
FA terminates as the superior labial artery (Fig. 4)	Type IV
FA terminates distal to the superior labial artery near NLF (Fig. 5)	Type V
FA terminates as the inferior alar artery (Fig. 6)	Type VI
The superior and inferior alar arteries, a branch of the FA, arise from the superior labial artery	
Without superior or inferior labial branches of the FA, the superior and inferior alar branches arise from the FA as a common trunk	
The superior and inferior labial branches of FA arise from a common trunk and terminate early	
FA terminates as the lateral nasal artery (Fig. 7)	Type VII
FA terminates as the angular artery (Fig. 8)	Type VIII
The angular type of FA extends and branches to the frontal region	
After the main trunk of the superior labial artery, FA divides into two branches, and a duplex angular artery is observed	
The type in which FA is hypoplastic and its remaining portion is supplied by a dominant transverse FA (Fig. 9)	Type IX
FA arises from the external carotid artery at a higher level than normal and courses transversely on the face (transverse type) (Fig. 10)	Type X
*Location of the FA relative to the NLF (FA–NLF Type)*	
Medial to NLF (Fig. 11e)	Medial
Lateral to NLF (Fig. 11f)	Lateral
Crossing NLF from lateral to medial (Fig. 11g)	Cross laterally
Crossing NLF from medial to lateral (Fig. 11h)	Cross medially
*FV Types*	
FV passes through the mandible at the anterior margin of the masseter muscle and opens into the retromandibular vein (Fig. 12a)	Type I
FV passes through the surface of the masseter muscle and opens into the superficial temporal vein (Fig. 12b)	Type II
FV passes through the surface of the masseter muscle and anastomoses with a branch from the superficial temporal vein (Fig. 12c)	Type III

*FA* facial artery, *FV* facial vein, *NLF* nasolabial fold

### Measurements

**Fig. 13:**
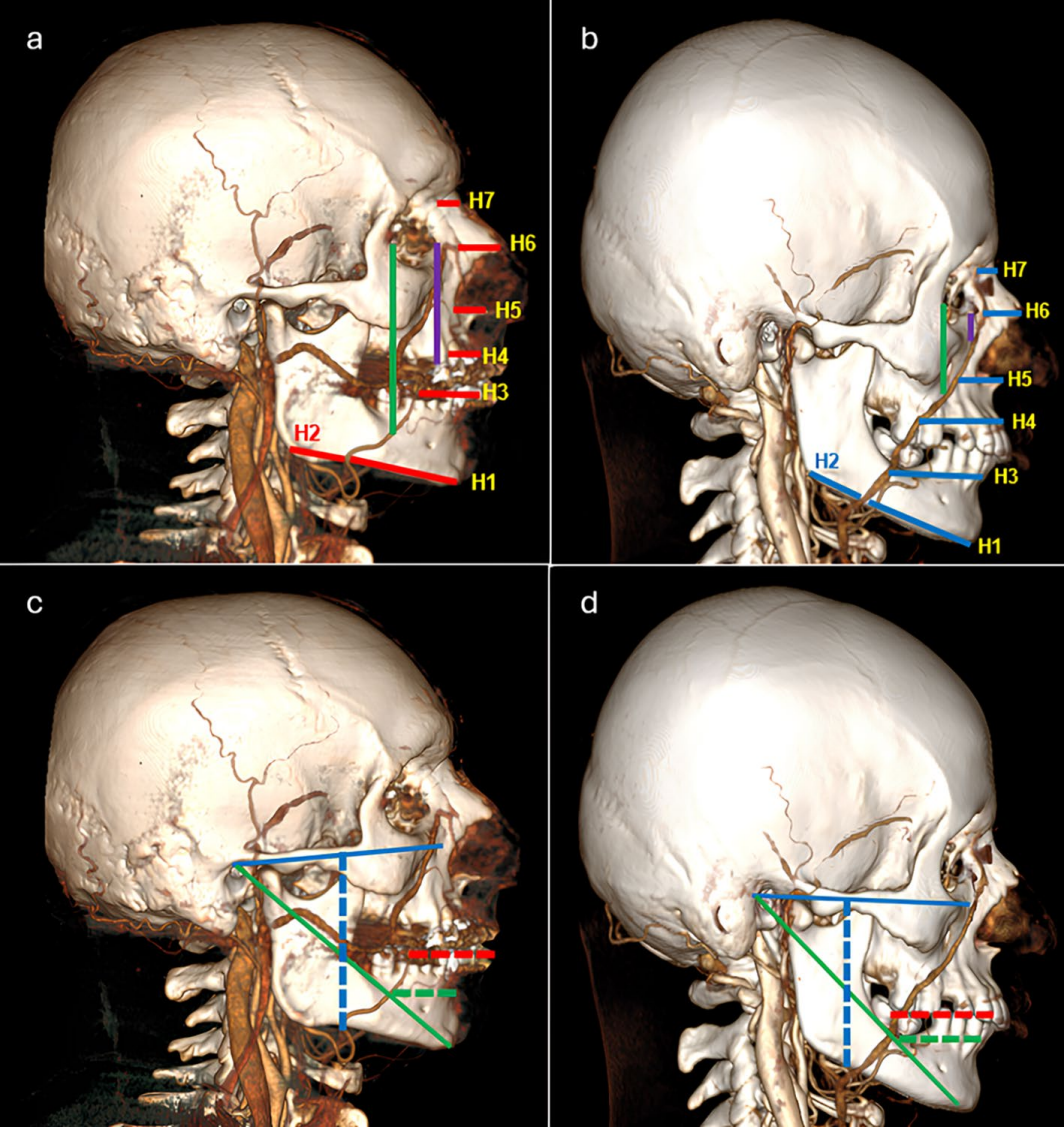
Distances of the FA and FV to anatomical landmarks and planes on three-dimensional volume rendering computed tomography angiography images. Distances of the FA (**a**) and FV (**b**) to the mental prominence (H1_MP_), mandibular angle (H2_MA_), alveolar process of the mandible (H3_APMDB_), alveolar process of the maxilla (H4_APMX_), anterior nasal spine (H5_ANS_), zygomatic arch (H6_ZA_), rhinion (H7_R_), nasion (H8_N_), medial orbital rim (purple line), and lateral orbital rim (green line). Distance between the FA (**c**) and FV (**d**) and the Frankfurt horizontal plane (blue dashed line), occlusal plane (red dashed line), and mandibular plane (green dashed line)

On 3D reconstructions, distances from the FA and FV to palpable bony landmarks and facial planes were measured. Horizontal measurements included mental protuberance (H1_MP_), mandibular angle (H2_MA_), alveolar process of mandible (H3_APMDB_), alveolar process of maxilla (H4_APMX_), anterior nasal spine (H5_ANS_), zygomatic arch (H6_ZA_), rhinion (H7_R_), nasion (H8_N_), while vertical distances were assessed from the medial (V1_MOR_) and lateral orbital rims (V2_LOR_). Additional measurements included distances from the Frankfurt horizontal plane to the mandibular exit point of the FA and FV (P1_FHP_) and from the occlusal (P2_OP_) and mandibular planes (P3_MDBP_) to their midline intersection (Fig. 13).

### Statistical Analysis

Statistical analyses were performed using SPSS 27.0 (IBM Corp., Armonk, NY, USA). Inter-observer reliability was excellent (ICC = 0.976). Normality was confirmed by skewness and kurtosis. Paired sample *t*-tests were used to compare right and left sides, while independent sample t-tests assessed gender-based differences. Chi-square tests were applied for categorical data. One-way ANOVA with Bonferroni and Tukey post hoc tests was used for comparisons involving more than two groups. Pearson correlation analysis was conducted to evaluate relationships between continuous variables, and statistical significance was set at *p* < 0.05. Effect sizes (*η*^2^ for ANOVA and Cohen’s d for paired and independent *t*-tests) were reported with 95% confidence intervals.

## Results

### Type of FA

We identified 9 of the 14 FA types reported in the literature and described one previously unreported variation, termed the transverse type (Type X) (Fig. 10). This variant was observed on the left side of a 30-year-old male, while the contralateral side showed a Type IX FA with a double artery (Fig. 9); therefore, this patient was excluded from all analyses except FA classification.

**Fig. 14:**
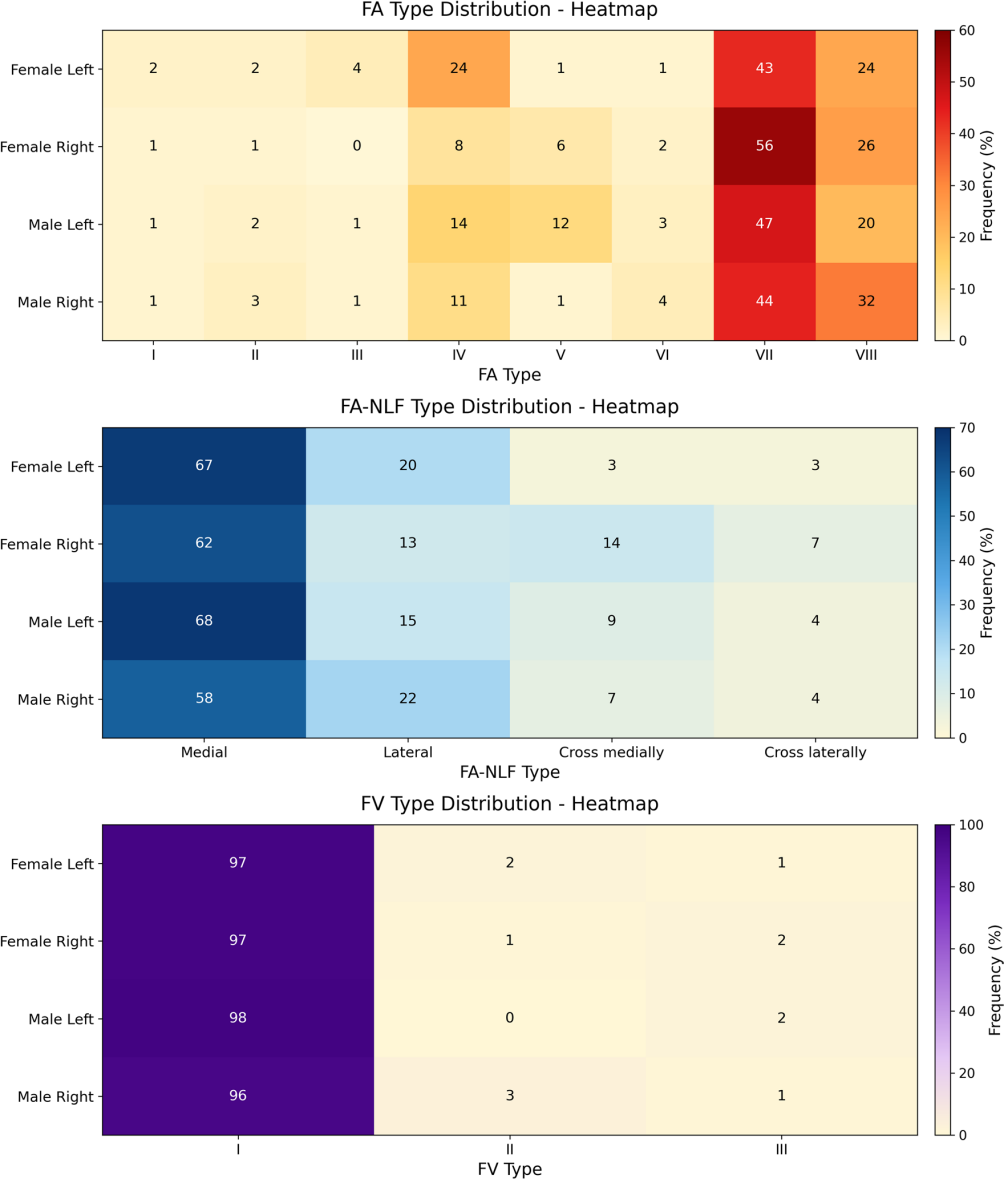
Color-coded heatmaps demonstrate the distribution frequencies of FA, FA–NLF, and FV types according to sex (female, male) and side (right, left). Each color intensity represents relative frequency (%)

The remaining 400 FA types were distributed as follows: Type I in 5, Type II in 8, Type III in 8, Type IV in 57, Type V in 20, Type VI in 10, Type VII in 190, and Type VIII in 102. Type VII was the most common on both sides in females and males, followed by Type VIII and Type IV, respectively. No Type III was found in FA on the right side of any female (Fig. 14). There was no significant difference among right-side FA types (*p *= 0.108, *χ*^2 ^= 11.772), but a significant difference was found on the left side (*p *= 0.038, *χ*^2 ^= 14.814).

### Location of the FA Relative to the NLF

The medial type of FA relative to the NLF was the most common on both sides in both genders, followed by lateral and cross medially types (Fig. 14). No significant difference was observed between the right (*p *= 0.140) and left (*p *= 0.282) sides in terms of lateralization.

### Course of the FV

Besides the two FV types reported in the literature, our study identified a third type. Type I was the most common on both sides, seen in 97% of females and 96% (right) and 98% (left) of males. Types II and III were equally common in both genders, with no significant difference by side (Fig. 14) (right: *p *= 0.512, left: *p *= 0.311).

### Morphometric Measurements in FA, FA–NLF, and FV Types

Morphometric comparisons among the eight FA types revealed significant post hoc differences in several parameters (Table 2). Type III showed distinct values—higher in right FA-H1_MP_ and FA-V1_MOR_, and lower in left FA-H4_APMX_, FA-P1_FHP_, FA-P2_OP_, and FA-V2_LOR_—than several other types.

**Table 2:** Comparative analysis of right- and left-side FA morphometric measurements according to FA types

Parameter (cm)		Type I (*n*=5) (mean±SD)	Type II (*n*=8) (mean±SD)	Type III (*n*=8) (mean±SD)	Type IV (*n*=57) (mean±SD)	Type V (*n*=20) (mean±SD)	Type VI (*n*=10) (mean±SD)	Type VII (*n*=190) (mean±SD)	Type VIII (*n*=102) (mean±SD)	*P* value	*η*^2^ [95% CI]
H1_MP_	R	4.50±1.24	5.02±0.57	5.94±0.55	4.76±0.60	4.40±0.61	5.32±0.99	4.80±0.57	4.83±0.70	0.008*	0.09 [0.00, 0.14]
	L	4.87±0.70	5.10±0.61	4.71±0.39	4.78±0.64	5.21.25±	5.95±1.21	4.99±0.69	4.98±0.76	0.132	0.06 [0.00, 0.10]
H2_MA_	R	4.45±0.70	5.25±0.42	4.93±0.51	4.42±0.52	4.73±0.41	4.28±0.65	4.47±0.69	4.54±0.82	0.366	0.03 [0.00, 0.06]
	L	4.48±0.43	4.71±0.09	4.87±0.82	4.72±0.65	5.03±0.31	5.31±1.15	4.55±0.65	4.76±0.76	0.074	0.05 [0.00, 0.09]
H3_APMDB_	R	2.26		2.98±0.25	2.64±0.43	2.59±0.44	2.77±0.58	2.77±0.56	2.95±0.68	0.272	0.03 [0.00, 0.07]
	L	2.36	2.66±0.01	2.94±0.55	2.69±0.40	2.98±0.26	3.37±0.94	2.80±0.58	3.09±0.55	<0.001*	0.09 [0.00, 0.14]
H4_APMX_	R			2.28±0.42	2.24±0.56	2.33±0.54	2.06±0.59	2.23±0.76	2.36±0.69	0.861	0.01 [0.00, 0.02]
	L			1.55±0.01	2.07±0.63	1.93±0.90	2.70±0.82	2.06±0.71	2.33±0.66	<0.001*	0.05 [0.00, 0.10]
H5_ANS_	R						1.59±0.06	1.56±0.75	1.63±0.61	0.852	0.00 [0.00, 0.02]
	L						1.58±0.30	1.40±0.58	1.68±0.69	0.054	0.04 [0.00, 0.11]
H6_ZA_	R							4.18±0.66	3.98±0.79	0.104	0.01 [0.00, 0.07]
	L							4.17±0.60	4.36±0.70	0.107	0.02 [0.00, 0.09]
H7_R_	R							1.64±0.42	1.50±0.44	0.058	0.02 [0.00, 0.10]
	L							1.55±0.40	1.57±0.43	0.854	0.00 [0.00, 0.03]
H8_N_	R							0.79±0.11	0.63±0.16	0.114	0.04 [0.00, 0.17]
	L								0.67±0.18		
V1_MOR_	R			4.63±0.15	3.65±1.21		3.10±1.24	2.20±1.05	1.83±0.89	<0.001*	0.24 [0.11, 0.34]
	L		2.93±0.01	4.55±001	3.86±1.01		3.33±0.56	2.26±1.13	1.97±1.05	0.002*	0.22 [0.07, 0.31]
V2_LOR_	R	7.40±0.12	7.10±0.21	6.77±0.65	7.15±0.61	7.37±0.51	7.50±0.57	7.58±0.89	7.59±1.01	0.324	0.04 [0.00, 0.07]
	L	7.74±0.22	8.74±0.58	6.21±0.08	7.17±0.63	8.32±1.16	7.33±0.54	7.70±0.91	7.80±0.93	<0.001*	0.17 [0.06, 0.24]
P1_FHP_	R	6.45±0.14	6.64±0.42	6.71±0.34	6.74±0.40	7.18±0.33	7.02±0.51	6.93±0.63	7.08±0.62	0.020*	0.04 [0.00, 0.08]
	L	6.50±0.45	6.93±0.76	6.55±0.02	6.64±0.49	7.33±0.32	7.95±0.80	7.03±0.69	6.96±0.59	<0.001*	0.13 [0.03, 0.19]
P2_OP_	R			2.71±0.06	2.72±0.52	3.16±0.08	2.65±0.41	2.83±0.71	2.94±0.93	<0.001*	0.01 [0.00, 0.04]
	L			2.06±0.20	2.64±0.58	2.99±0.20	2.78±0.56	2.72±0.52	2.96±0.87	<0.001*	0.06 [0.00, 0.12]
P3_MDBP_	R	2.63		3.22±0.54	2.96±0.68	3.11±0.58	2.54±0.53	3.13±0.64	3.55±0.93	0.024*	0.10 [0.01, 0.16]
	L	2.63	2.93±0.06	2.87±0.71	2.93±0.56	3.53±0.30	3.22±1.16	3.13±0.54	3.42±0.79	<0.001*	0.09 [0.00, 0.15]

^*^Statistically significant *p* value, *η*^*2*^ eta squared, *CI* confidence interval, *SD* standard deviation,* n* sample size,* R* right, *L* left, *FA* facial artery, *H1*_*MP*_ mental protuberance, *H2*_*MA*_ mandibular angle, *H3*_*APMDB*_ alveolar process of mandible, *H4*_*APMX*_ alveolar process of maxilla, *H5*_*ANS*_ anterior nasal spine, *H6*_*ZA*_ zygomatic arch, *H7*_*R*_ rhinion, *H8*_*N*_ nasion, *V1*_*MOR*_ medial orbital rim, *V2*_*LOR*_ lateral orbital rim, *P1*_*FHP*_ Frankfurt horizontal plane, *P2*_*OP*_ occlusal plane, *P3*_*MDBP*_ mandibular plane

FA measurements by NLF types (Table 3) showed significant right-sided differences in FA-H2_MA_, FA-H4_APMX_, FA-H5_ANS_, FA-P1_FHP_, and FA-P2_OP_ (*p *< 0.005), with most values higher in the lateral type.

**Table 3:** Comparative analysis of right- and left-side morphometric measurements according to FA–NLF types and FV types

Parameter (cm)		FA–NLF						FV				
		Medial (*n*=255) (mean±SD)	Lateral (*n*=70) (mean±SD)	Cross medially (*n*=33) (mean±SD)	Cross laterally (*n*=18) (mean±SD)	*P* value	*η*^2^ [95% CI]	Type I (*n*=388) (mean±SD)	Type II (n=6) (mean±SD)	Type III (n=6) (mean±SD)	*P* value	*η*^2^ [95% CI]
H1_MP_	R	4.73±0.61	5.13±0.70	4.82±0.58	4.51±0.55	0.005*	0.06 [0.00, 0.13]	5.25±0.75		5.27±0.22	0.967	0.00 [0.00, 0.00]
	L	4.99±0.72	4.93±0.56	5.23±1.03	4.65±0.32	0.117	0.01 [0.00, 0.05]	5.41±0.77		5.88±0.57	0.301	0.00 [0.00, 0.04]
H2_MA_	R	4.46±0.69	4.84±0.54	4.51±0.78	3.71±0.65	<0.001*	0.11 [0.03, 0.19]	3.38±0.85		4.05±0.27	0.177	0.09 [0.00, 0.05]
	L	4.68±0.70	4.74±0.55	4.92±0.60	3.98±0.76	0.030*	0.04 [0.00, 0.10]	3.51±0.85		4.39±0.84	0.080	0.01 [0.00, 0.06]
H3_APMDB_	R	2.80±0.62	2.98±0.58	2.76±0.47	2.40±0.46	0.041*	0.04 [0.00, 0.10]	3.77±0.86		4.62±0.32	0.095	0.01 [0.00, 0.06]
	L	2.86±0.58	2.89±0.51	3.02±0.50	2.71±0.36	0.698	0.08 [0.00, 0.03]	3.86±0.90		4.68±0.71	0.120	0.01 [0.00, 0.05]
H4_APMX_	R	2.23±0.77	2.67±0.52	2.15±0.35	1.68±0.44	<0.001*	0.10 [0.02, 0.18]	3.45±0.86	4.58±0.07	4.17±0.61	0.014*	0.04 [0.00, 0.10]
	L	2.07±0.72	2.36±0.75	2.25±0.42	1.81±0.53	0.096	0.03 [0.00, 0.08]	3.48±0.96	4.59±0.01	3.87±0.69	0.219	0.01 [0.00, 0.05]
H5_ANS_	R	1.53±0.64	2.24±0.75	1.11±0.29	1.43±0.51	<0.001*	0.22 [0.10, 0.31]	2.23±0.77	2.49±0.23	2.64±0.69	0.537	0.00 [0.00, 0.03]
	L	1.46±0.53	1.98±0.96	1.28±0.27	1.06±0.17	<0.001*	0.12 [0.02, 0.21]	2.33±0.72	2.45±0.09	2.31±0.77	0.974	0.00 [0.00, 0.00]
H6_ZA_	R	4.05±0.74	4.41±0.56	4.27±0.48	3.11±0.59	<0.001*	0.13 [0.03, 0.22]	3.61±0.49	4.36±0.22	4.14±0.22	0.003*	0.05 [0.00, 0.12]
	L	4.24±0.64	4.31±0.53	4.41±0.60	3.66±0.86	0.078	0.05 [0.00, 0.12]	3.62±0.50	4.47±0.02	4.13±0.23	0.017*	0.04 [0.00, 0.10]
H7_R_	R	1.54±0.36	1.80±0.61	1.62±0.33	1.20±0.12	<0.001*	0.10 [0.01, 0.19]	1.61±0.44	1.39±0.26	1.68±0.10	0.586	0.00 [0.00, 0.03]
	L	1.53±0.35	1.80±0.63	1.55±0.35	1.32±0.32	0.230	0.07 [0.00, 0.17]	1.63±0.40	1.24±0.02	1.63±0.02	<0.001*	0.00 [0.00, 0.04]
H8_N_	R	0.63±0.18	0.62±0.14	0.73±0.04	0.74±0.11	0.376	0.05 [0.00, 0.16]	0.72±0.24	0.82±0.07	0.84±0.05	0.545	0.00 [0.00, 0.03]
	L	0.65±0.18	0.65±0.21	0.65±0.11	0.79±0.01	0.701	0.03 [0.00, 0.13]	0.71±0.22	0.71±0.06	0.81±0.03	0.761	0.00 [0.00, 0.02]
V1_MOR_	R	2.05±1.14	1.85±0.64	2.44±0.95	2.86±0.54	<0.001*	0.06 [0.00, 0.13]	1.10±0.44	1.54±0.14	1.12±0.35	0.141	0.02 [0.00, 0.06]
	L	2.30±1.23	1.91±1.03	2.19±0.47	2.92±0.75	0.101	0.03 [0.00, 0.10]	0.94±0.32	1.63±0.06	1.12±0.35	0.009*	0.04 [0.00, 0.11]
V2_LOR_	R	7.32±0.82	7.67±0.74	7.74±0.80	9.07±0.61	<0.001*	0.22 [0.11, 0.31]	6.50±1.08	6.17±0.71	5.74±1.24	0.416	0.00 [0.00, 0.04]
	L	7.58±0.96	7.73±0.66	7.74±0.74	8.53±1.22	0.230	0.04 [0.00, 0.09]	6.69±1.23	6.58±0.08	5.70±1.28	0.385	0.01 [0.00, 0.04]
P1_FHP_	R	6.88±0.61	7.15±0.48	6.72±0.48	7.83±0.14	<0.001*	0.16 [0.07, 0.25]	6.62±0.56	5.07±1.27	7.08±0.28	<0.001*	0.13 [0.05, 0.21]
	L	6.93±0.69	1.07±0.49	6.85±0.40	7.59±0.68	0.067	0.04 [0.00, 0.10]	6.63±0.70	4.42±0.02	7.03±0.31	<0.001*	0.09 [0.02, 0.17]
P2_OP_	R	2.81±0.78	3.26±0.68	2.88±0.43	2.17±0.45	<0.001*	0.10 [0.02, 0.18]	3.84±0.89	4.58±0.86	4.86±0.69	0.056	0.02 [0.00, 0.08]
	L	2.68±0.56	3.14±0.78	3.05±0.21	2.37±0.53	<0.001*	0.10 [0.02, 0.18]	3.93±0.98	4.68±0.07	3.85±0.40	0.552	0.00 [0.00, 0.03]
P3_MDBP_	R	3.20±0.80	3.54±0.82	3.15±0.44	2.62±0.28	<0.001*	0.07 [0.00, 0.13]	3.93±0.82		4.45±0.31	0.275	0.00 [0.00, 0.04]
	L	3.09±0.65	3.53±0.60	3.23±0.16	3.17±0.55	0.013*	0.06 [0.07, 0.13]	4.03±0.83		4.26±0.02	0.626	0.00 [0.00, 0.02]

^*^Statistically significant p value, *η*^*2*^ eta squared, *CI* confidence interval, *SD* standard deviation,* n* sample size,* R* right, *L* left, *FA-NLF* location of the facial artery relative to the nasolabial fold, *FV* facial vein, *H1*_*MP*_ mental protuberance, *H2*_*MA*_ mandibular angle, *H3*_*APMDB*_ alveolar process of mandible, *H4*_*APMX*_ alveolar process of maxilla, *H5*_*ANS*_ anterior nasal spine, *H6*_*ZA*_ zygomatic arch, *H7*_*R*_ rhinion, *H8*_*N*_ nasion, *V1*_*MOR*_ medial orbital rim, *V2*_*LOR*_ lateral orbital rim, *P1*_*FHP*_ Frankfurt horizontal plane, *P2*_*OP*_ occlusal plane, *P3*_*MDBP*_ mandibular plane

For FV (Table 3), all significant measurements except FV-H7_R_ and FV-P1_FHP_ were higher in Type II. FV-H7_R_ and FV-P1_FHP_ differed significantly between Types I–II and II–III (*p* = 0.000).

### Lateralization-Related Differences in Morphometric Measurements

Significant right–left differences were observed in selected FA (H1_MP_, H2_MA_, H4_APMX_, V2_LOR_) and FV (H1_MP_, H2_MA_, V1_MOR_, V2_LOR_) parameters (*p *< 0.05), all with small effect sizes, indicating minimal asymmetry and overall bilateral symmetry (Table 4).

**Table 4:** Comparison of FA- and FV-based morphometric measurements between right and left sides

Parameter (cm)	Right (mean ± SD)	Left (mean ± SD)	Mean difference ± SD	*t*	*P* value	Cohen’s d [95% CI]
*FA*						
H1_MP_	4.83 ± 0.66	4.97 ± 0.70	− 0.15 ± 0.49	− 4.18	<0.001*	− 0.29 [− 0.44, − 0.15]
H2_MA_	3.52 ± 0.71	3.68 ± 0.68	− 0.16 ± 0.79	− 2.97	0.003*	− 0.21 [− 0.35, − 0.07]
H3_APMDB_	2.81 ± 0.59	2.86 ± 0.55	− 0.05 ± 0.66	− 1.04	0.298	− 0.08 [− 0.22, 0.07]
H4_APMX_	2.28 ± 0.71	2.12 ± 0.71	0.16 ± 0.95	2.27	0.024*	0.17 [0.02, 0.31]
H5_ANS_	1.58 ± 0.64	1.51 ± 0.65	0.07 ± 0.79	0.99	0.323	0.09 [− 0.09, 0.27]
H6_ZA_	4.19 ± 0.72	4.27 ± 0.63	− 0.09 ± 0.82	− 1.10	0.275	− 0.11 [− 0.30, 0.08]
H7_R_	1.52 ± 0.38	1.59 ± 0.42	− 0.07 ± 0.55	− 1.04	0.303	− 0.12 [− 0.35, 0.11]
H8_N_	0.67 ± 0.20	0.69 ± 0.21	− 0.02 ± 0.34	− 0.26	0.801	− 0.06 [− 0.54, 0.41]
V1_MOR_	2.25 ± 1.14	2.33 ± 1.23	− 0.09 ± 1.55	− 0.55	0.581	− 0.06 [− 0.25, 0.14]
V2_LOR_	7.47 ± 0.88	7.66 ± 0.94	− 0.19 ± 1.12	− 2.36	0.019*	− 0.17 [− 0.32, − 0.03]
P1_FHP_	6.95 ± 0.60	6.96 ± 0.64	− 0.01 ± 0.62	− 0.16	0.870	− 0.01 [− 0.15, 0.13]
P2_OP_	2.87 ± 0.75	2.76 ± 0.62	0.10 ± 0.92	1.54	0.124	0.11 [− 0.03, 0.26]
P3_MDBP_	3.22 ± 0.76	3.19 ± 0.64	0.03 ± 0.80	0.54	0.589	0.04 [− 0.10, 0.18]
*FV*						
H1_MP_	5.26 ± 0.75	5.41 ± 0.77	− 0.15 ± 0.55	− 3.86	<0.001*	− 0.28 [− 0.42, − 0.13]
H2_MA_	4.39 ± 0.85	4.51 ± 0.86	− 0.12 ± 0.79	− 2.15	0.033*	− 0.15 [− 0.30, − 0.01]
H3_APMDB_	3.79 ± 0.86	3.86 ±0.90	− 0.07 ± 0.83	− 1.14	0.257	− 0.08 [− 0.22, 0.06]
H4_APMX_	3.49 ± 0.86	3.50 ± 0.96	− 0.02 ± 1.04	− 0.21	0.834	− 0.01 [− 0.15, 0.12]
H5_ANS_	2.24 ± 0.76	2.33 ± 0.72	− 0.09 ± 0.74	− 1.67	0.097	− 0.12 [− 0.26, 0.02]
H6_ZA_	3.63 ± 0.50	3.64 ± 0.51	− 0.01 ± 0.64	− 0.15	0.882	− 0.01 [− 0.15, 0.13]
H7_R_	1.61 ± 0.43	1.62 ± 0.39	− 0.01 ± 0.49	− 0.43	0.670	− 0.03 [− 0.17, 0.11]
H8_N_	0.73 ± 0.24	0.71 ± 0.22	0.02 ± 0.29	0.87	0.384	0.06 [− 0.08, 0.21]
V1_MOR_	1.11 ± 0.43	0.95 ± 0.33	0.16 ± 0.54	4.06	<0.001*	0.29 [0.15, 0.44]
V2_LOR_	6.48 ± 1.08	6.67 ± 1.23	− 0.19 ± 1.26	− 2.19	0.030*	− 0.15 [− 0.29, − 0.02]
P1_FHP_	6.60 ± 0.62	6.61 ± 0.73	− 0.01 ± 0.80	− 0.23	0.819	− 0.02 [− 0.15, 0.12]
P2_OP_	3.87 ± 0.89	3.93 ± 0.97	− 0.06 ± 1.01	− 0.89	0.375	− 0.06 [− 0.20, 0.08]
P3_MDBP_	3.94 ± 0.82	4.03 ± 0.83	− 0.08 ± 0.99	− 1.20	0.233	− 0.09 [− 0.23, 0.06]

^*^Statistically significant p value,* t* Student’s t statistic, *Cohen’s d* effect size, *CI* confidence interval, *SD* standard deviation,* n* sample size,* R* right, *L* left, *FA* facial artery, *FV* facial vein, *H1*_*MP*_ mental protuberance, *H2*_*MA*_ mandibular angle, *H3*_*APMDB*_ alveolar process of mandible, *H4*_*APMX*_ alveolar process of maxilla, *H5*_*ANS*_ anterior nasal spine, *H6*_*ZA*_ zygomatic arch, *H7*_*R*_ rhinion, *H8*_*N*_ nasion, *V1*_*MOR*_ medial orbital rim, *V2*_*LOR*_ lateral orbital rim, *P1*_*FHP*_ Frankfurt horizontal plane, *P2*_*OP*_ occlusal plane, *P3*_*MDBP*_ mandibular plane

### Gender-Related Differences in Morphometric Measurements

**Fig. 15:**
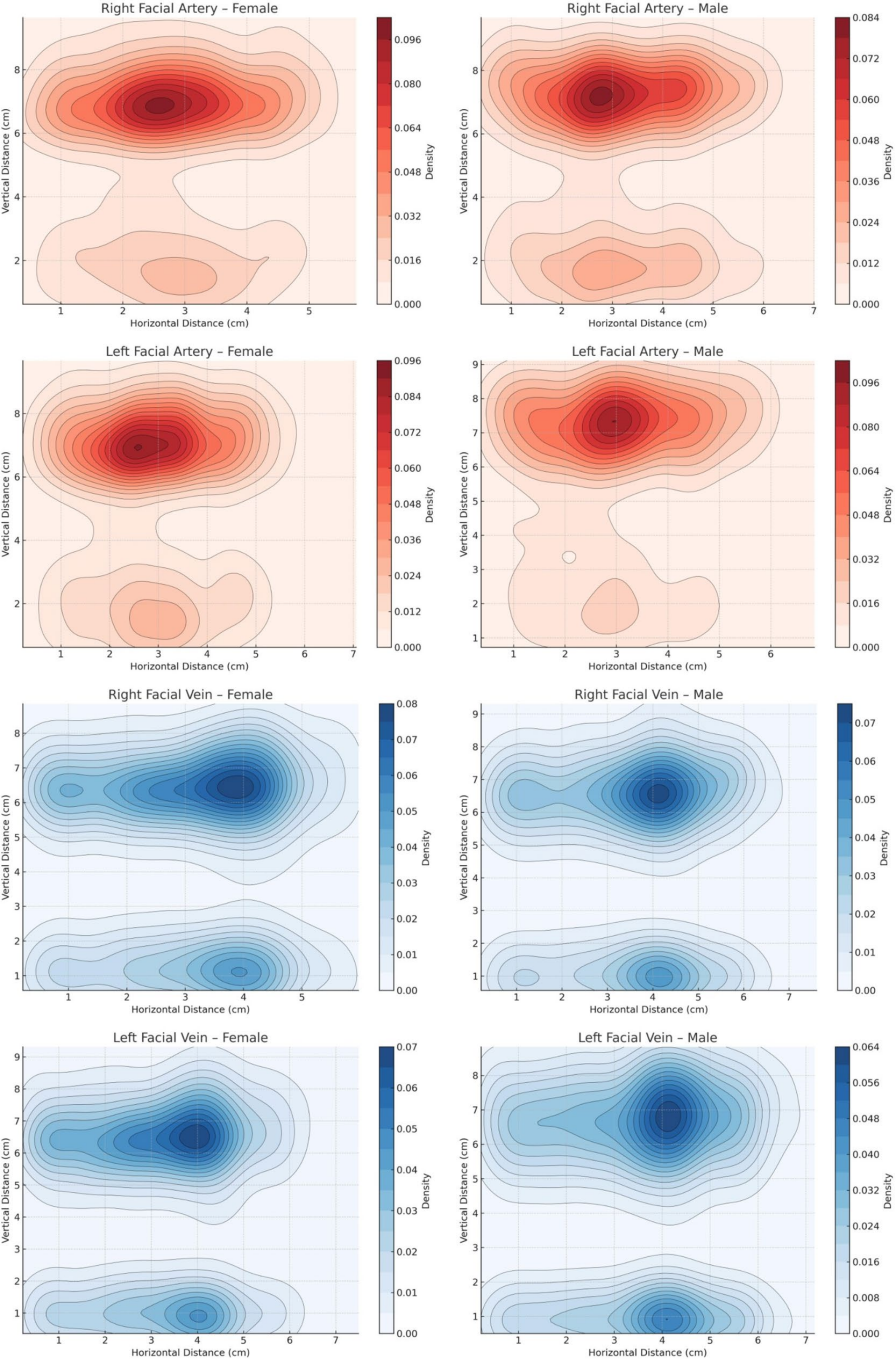
Density maps prepared using two-dimensional Gaussian Kernel Density Estimation to visualize the distribution density of the right and left FA and FV in male and female individuals. The color gradient represents vascular density: dark red indicates regions of highest FA density, while light red denotes areas of lower FA density; similarly, dark blue indicates to regions of highest FV density, whereas light blue reflects areas of lower FV density

All statistically significant FA and FV measurements were greater in males (Table 5). Density maps demonstrated broader and more defined vascular distributions in males, with marked right–left symmetry in both sexes (Fig. 15). While the highest vertical density for both FA and FV was observed at the P1_FHP_, the densest regions in the horizontal plane were as follows:

**Table 5:** Comparison of FA and FV-based morphometric measurements between right and left sides in female and male

Parameter (cm)		FA						FV					
		Female (*n*=100) (mean±SD)		Male (*n*=100) (mean±SD)	*t*	*P* value	Cohen’s d [95% CI]	Female (*n*=100) (mean±SD)		Male (*n*=100) (mean±SD)	*t*	*P* value	Cohen’s d [95% CI]
H1_MP_	R	4.54±0.46		5.12±0.70	− 6.86	<0.001*	− 0.97 [− 1.26, − 0.67]	4.96±0.59		5.56±0.78	− 6.03	<0.001*	− 0.86 [− 1.15, − 0.56]
	L	4.80±0.68		5.15±0.67	− 3.61	<0.001*	− 0.51 [− 0.79, − 0.22]	5.21±0.78		5.62±0.70	− 3.82	<0.001*	− 0.54 [− 0.82, − 0.25]
H2_MA_	R	4.06±0.79		4.72±0.77	− 5.59	<0.001*	− 0.79 [− 1.07, − 0.50]	3.25±0.60		3.78±0.71	− 5.84	<0.001*	− 0.83 [− 1.12, − 0.54]
	L	4.31±0.89		4.73±0.76	− 2.44	<0.001*	− 0.34 [− 0.62, − 0.06]	3.56±0.67		3.80±0.66	− 3.57	0.015*	− 0.50 [− 0.79, − 0.22]
H3_APMDB_	R	2.64±0.49		2.97±0.63	− 4.05	<0.001*	− 0.58 [− 0.86, − 0.29]	3.53±0.78		4.05±0.86	− 4.43	<0.001*	− 0.63 [− 0.91, − 0.34]
	L	2.84±0.58		2.89±0.52	− 0.53	0.592	− 0.07 [− 0.35, 0.20]	3.68±0.92		4.00±0.84	− 2.94	0.004*	− 0.41 [− 0.69, − 0.13]
H4_APMX_	R	2.11±0.55		2.43±0.81	− 3.25	<0.001*	− 0.46 [− 0.75, − 0.18]	3.24±0.80		3.73±0.86	− 4.12	<0.001*	− 0.58 [− 0.86, − 0.30]
	L	2.09±0.63		2.14±0.78	− 0.44	0.654	− 0.06 [− 0.34, − 0.21]	3.36±0.96		3.64±0.93	− 2.06	0.040*	− 0.29 [− 0.57, − 0.01]
H5_ANS_	R	1.45±0.65		1.73±0.71	− 2.56	0.011*	− 0.40 [− 0.71, − 0.09]	2.01±0.63		2.47±0.82	− 4.43	<0.001*	− 0.62 [− 0.91, − 0.34]
	L	1.58±0.70		1.42±0.53	1.47	0.147	0.25 [− 0.08, − 0.59]	2.16±0.71		2.50±0.69	− 3.36	<0.001*	− 0.47 [− 0.75, − 0.19]
H6_ZA_	R	3.96±0.67		4.25±0.73	− 2.46	0.015*	− 0.40 [− 0.72, − 0.07]	3.52±0.45		3.74±0.52	− 3.14	0.002*	− 0.44 [− 0.72, − 0.16]
	L	4.10±0.61		4.37±0.65	− 2.47	0.015*	− 0.43 [− 0.78, − 0.08]	3.56±0.50		3.72±0.51	− 2.29	0.023*	− 0.32 [− 0.60, − 0.04]
H7_R_	R	1.50±0.42		1.64±0.44	− 1.81	0.073	− 0.32 [− 0.67, − 0.03]	1.53±0.39		1.69±0.45	− 2.52	0.012*	− 0.35 [− 0.63, − 0.07]
	L	1.57±0.44		1.54±0.38	0.37	0.706	0.07 [− 0.31, 0.47]	1.56±0.39		1.70±0.40	− 2.59	0.010*	− 0.36 [− 0.64, − 0.08]
H8_N_	R	0.65±0.17		0.63±0.16	0.36	0.717	0.09 [− 0.41, 0.61]	0.69±0.23		0.77±0.25	− 2.40	0.017*	− 0.34 [− 0.63, − 0.06]
	L	0.65±0.16		0.68±0.20	− 0.50	0.616	− 0.15 [− 0.75, − 0.44]	0.69±0.21		0.73±0.23	− 1.30	0.194	− 0.18 [− 0.46, − 0.09]
V1_MOR_	R	2.18±1.17		2.22±1.09	− 0.23	0.815	− 0.03 [− 0.36, 0.28]	1.18±0.47		1.00±0.38	2.40	0.017*	0.34 [0.06, 0.62]
	L	2.16±1.13		2.57±1.22	− 1.90	0.060	− 0.24 [− 0.52, − 0.03]	1.00±0.38		0.90±0.26	2.04	0.042*	0.29 [0.01, 0.57]
V2_LOR_	R	7.41±0.90		7.59±0.85	− 1.41	0.158	− 0.20 [− 0.48, 0.07]	6.35±1.02		6.61±1.13	− 1.70	0.090	− 0.24 [− 0.51, 0.03]
	L	7.49±0.86		7.82±0.99	− 2.48	0.014*	− 0.35 [− 0.64, − 0.07]	6.55±1.10		6.80±1.35	− 1.42	0.156	− 0.20 [− 0.47, 0.07]
P1_FHP_	R	6.84±0.57		7.06±0.61	− 2.62	0.009*	− 0.37 [− 0.65, − 0.09]	6.52±0.59		6.68±0.63	− 1.80	0.073	− 0.25 [− 0.53, 0.02]
	L	6.88±0.58		7.03±0.70	− 1.72	0.086	− 0.24 [− 0.52, 0.03]	6.51±0.72		6.71±0.73	− 1.98	0.049*	− 0.28 [− 0.55, − 0.00]
P2_OP_	R	2.71±0.63		3.01±0.82	− 2.85	0.005*	− 0.41 [− 0.69, − 0.12]	3.60±0.83		4.14±0.87	− 4.49	<0.001*	− 0.63 [− 0.92, − 0.35]
	L	2.73±0.63		2.80±0.62	− 0.85	0.394	− 0.12 [− 0.40, 0.16]	3.68±0.96		4.19±0.91	− 3.81	<0.001*	− 0.54 [− 0.82, − 0.25]
P3_MDBP_	R	3.02±0.56		3.43±0.90	− 3.78	<0.001*	− 0.54 [− 0.83, − 0.25]	3.65±0.67		4.23±0.85	− 5.31	<0.001*	− 0.76 [− 1.04, − 0.46]
	L	3.13±0.63		3.22±0.64	− 0.97	0.332	− 0.14 [− 0.42, 0.14]	3.85±0.81		4.20±0.80	− 3.05	0.003*	− 0.43 [− 0.71, − 0.15]

^*^Statistically significant *p* value,* t* Student’s t statistic, *Cohen’s d* effect size, *CI* confidence interval, *SD* standard deviation,* n* sample size,* R* right, *L* left, *FA* facial artery, *FV* facial vein, *H1*_*MP*_ mental protuberance, *H2*_*MA*_ mandibular angle, *H3*_*APMDB*_ alveolar process of mandible, *H4*_*APMX*_ alveolar process of maxilla, *H5*_*ANS*_ anterior nasal spine, *H6*_*ZA*_ zygomatic arch, *H7*_*R*_ rhinion, *H8*_*N*_ nasion, *V1*_*MOR*_ medial orbital rim, *V2*_*LOR*_ lateral orbital rim, *P1*_*FHP*_ Frankfurt horizontal plane, *P2*_*OP*_ occlusal plane, *P3*_*MDBP*_ mandibular plane

- FA from H3_APMDB_:

Right: 2.61 cm in females; 2.80 cm in males

Left: 2.57 cm in females; 2.99 cm in males

- FV from H6_ZA_:

Right: 3.88 cm in females; 4.16 cm in males

Left: 3.97 cm in females; 4.10 cm in males

### Age-Related Differences in Morphometric Measurements

Mean right and left FA and FV measurements across age groups are presented in Tables 6 and 7, respectively. Post hoc tests showed significant FA differences only between the 60–69 group and others (*p *< 0.005), with FA-H1_MP_ and FA-H2_MA_ differing across all groups (*p* = 0.000). For FV, right-side measurements FV-H1_MP_, FV-H2_MA_, FV-H3_APMDB_, FV-H4_APMX_, FV-H5_ANS_, FV-P2_OP_, and FV-P3_MDBP_ showed statistically significant differences across all age groups, while left-side differences were significant only between the 60–69 group and others (*p *< 0.005).

**Table 6:** Comparative analysis of right- and left-side FA morphometric measurements according to age groups

Parameter (cm)		30-39 years (*n*=50) (mean±SD)	40-49 years (*n*=50) (mean±SD)	50-59 years (*n*=50) (mean±SD)	60-69 years (*n*=50) (mean±SD)	*P* value	*η*^2^ [95% CI]
H1_MP_	R	5.56±0.54	5.06±0.41	4.51±0.38	4.19±0.17	<0.001*	0.63 [0.55, 0.68]
	L	5.49±0.61	5.39±0.63	4.72±0.42	4.31±0.27	<0.001*	0.48 [0.38, 0.55]
H2_MA_	R	5.03±0.65	4.59±0.56	4.26±0.81	3.69±0.73	<0.001*	0.11 [0.03, 0.18]
	L	4.99±0.67	4.77±0.75	4.49±0.79	3.84±0.76	<0.001*	0.17 [0.08, 0.26]
H3_APMDB_	R	3.04±0.65	2.84±0.63	2.67±0.52	2.68±0.46	0.005*	0.06 [0.00, 0.12]
	L	2.97±0.52	3.09±0.63	2.74±0.45	2.67±0.48	<0.001*	0.09 [0.02, 0.16]
H4_APMX_	R	2.48±0.61	2.32±0.70	2.16±0.77	2.12±0.70	0.055	0.03 [0.00, 0.09]
	L	2.14±0.77	2.29±0.73	2.06±0.67	1.97±0.63	0.150	0.02 [0.00, 0.07]
H5_ANS_	R	1.61±0.73	1.52±0.52	1.55±0.69	1.68±0.82	0.750	0.00 [0.00, 0.03]
	L	1.45±0.53	1.54±0.54	1.41±0.55	1.59±0.84	0.636	0.01 [0.00, 0.05]
H6_ZA_	R	4.51±0.67	4.33±0.42	4.00±0.69	3.59±0.68	<0.001*	0.24 [0.11, 0.33]
	L	4.48±0.67	4.22±0.54	4.35±0.55	3.88±0.67	0.002*	0.11 [0.01, 0.20]
H7_R_	R	1.71±0.45	1.56±0.38	1.59±0.39	1.48±0.48	0.220	0.03 [0.00, 0.10]
	L	1.61±0.38	1.61±0.40	1.47±0.36	1.55±0.49	0.589	0.02 [0.00, 0.07]
H8_N_	R	0.67±0.15	0.58±0.09	0.69±0.19	0.63±0.17	0.394	0.05 [0.00, 0.15]
	L	0.66±0.19	0.63±0.11	0.62±0.16	0.71±0.21	0.661	0.04 [0.00, 0.14]
V1_MOR_	R	2.61±1.15	2.28±0.99	2.20±0.99	1.79±1.24	0.018*	0.06 [0.00, 0.14]
	L	2.77±0.98	2.31±0.97	2.42±1.26	1.90±1.34	0.039*	0.07 [0.00, 0.15]
V2_LOR_	R	7.59±0.75	7.58±0.83	7.47±0.88	7.37±1.05	0.572	0.01 [0.00, 0.04]
	L	7.89±0.87	7.80±0.92	7.58±1.00	7.36±0.89	0.024*	0.04 [0.00, 0.10]
P1_FHP_	R	7.11±0.65	7.04±0.57	6.86±0.58	6.79±0.55	0.030*	0.04 [0.00, 0.10]
	L	7.08±0.67	7.08±0.70	7.00±0.59	6.67±0.53	<0.001*	0.06 [0.00, 0.13]
P2_OP_	R	3.06±0.64	2.79±0.62	2.89±0.86	2.67±0.79	0.067	0.03 [0.00, 0.09]
	L	2.83±0.60	2.90±0.65	2.71±0.57	2.62±0.65	0.119	0.03 [0.00, 0.08]
P3_MDBP_	R	3.43±0.82	3.32±0.73	3.19±0.72	2.93±0.73	0.009*	0.05 [0.00, 0.12]
	L	3.36±0.60	3.37±0.64	3.13±0.51	2.82±0.63	<0.001*	0.12 [0.04, 0.20]

^*^Statistically significant p value, *η*^*2*^ eta squared, *CI* confidence interval, *SD* standard deviation,* n* sample size,* R* right, *L* left, *FA* facial artery, *H1*_*MP*_ mental protuberance, *H2*_*MA*_ mandibular angle, *H3*_*APMDB*_ alveolar process of mandible, *H4*_*APMX*_ alveolar process of maxilla, *H5*_*ANS*_ anterior nasal spine, *H6*_*ZA*_ zygomatic arch, *H7*_*R*_ rhinion, *H8*_*N*_ nasion, *V1*_*MOR*_ medial orbital rim, *V2*_*LOR*_ lateral orbital rim, *P1*_*FHP*_ Frankfurt horizontal plane, *P2*_*OP*_ occlusal plane, *P3*_*MDBP*_ mandibular plane

**Table 7:** Comparative analysis of right- and left-side FV morphometric measurements according to age groups

Parameter (cm)		30-39 years (*n*=50) (mean±SD)	40-49 years (*n*=50) (mean±SD)	50-59 years (*n*=50) (mean±SD)	60-69 years (*n*=50) (mean±SD)	*P* value	*η*^2^ [95% CI]
H1_MP_	R	5.97±0.59	5.54±0.49	5.06±0.56	4.48±0.38	<0.001*	0.54 [0.44, 0.60]
	L	5.96±0.63	5.74±0.66	5.30±0.56	4.67±0.49	<0.001*	0.41 [0.30, 0.49]
H2_MA_	R	3.81±0.84	3.68±0.65	3.33±0.59	3.25±0.57	<0.001*	0.33 [0.22, 0.42]
	L	3.96±0.73	3.96±0.64	3.50±0.55	3.31±0.53	<0.001*	0.25 [0.15, 0.34]
H3_APMDB_	R	4.52±0.63	3.99±0.58	3.69±0.73	2.97±0.67	<0.001*	0.42 [0.31, 0.50]
	L	4.35±0.81	4.19±0.82	3.83±0.69	3.12±0.75	<0.001*	0.27 [0.17, 0.36]
H4_APMX_	R	4.14±0.66	3.67±0.55	3.34±0.77	2.79±0.84	<0.001*	0.32 [0.21, 0.40]
	L	3.79±1.01	3.66±1.10	3.52±0.66	3.03±0.84	<0.001*	0.09 [0.02, 0.16]
H5_ANS_	R	2.75±0.71	2.42±0.51	2.22±0.70	1.58±0.61	<0.001*	0.31 [0.20, 0.39]
	L	2.72±0.68	2.57±0.61	2.22±0.52	1.81±0.69	<0.001*	0.23 [0.13, 0.32]
H6_ZA_	R	3.73±0.54	3.76±0.40	3.56±0.57	3.49±0.44	0.009*	0.05 [0.00, 0.10]
	L	3.64±0.54	3.67±0.44	3.71±0.53	3.55±0.51	0.436	0.01 [0.00, 0.04]
H7_R_	R	1.72±0.42	1.65±0.42	1.63±0.41	1.45±0.44	0.014*	0.05 [0.00, 0.11]
	L	1.76±0.36	1.75±0.33	1.58±0.38	1.41±0.41	<0.001*	0.12 [0.04, 0.20]
H8_N_	R	0.76±0.28	0.65±0.20	0.74±0.24	0.77±0.22	0.085	0.03 [0.00, 0.08]
	L	0.69±0.20	0.68±0.17	0.70±0.24	0.77±0.25	0.200	0.02 [0.00, 0.06]
V1_MOR_	R	1.19±0.48	1.07±0.31	1.11±0.49	1.07±0.43	0.464	0.01 [0.00, 0.04]
	L	0.87±0.22	0.91±0.25	1.00±0.40	1.01±0.39	0.088	0.03 [0.00, 0.08]
V2_LOR_	R	6.43±1.27	6.48±1.11	6.49±0.90	6.52±1.04	0.986	0.00 [0.00, 0.00]
	L	6.71±1.55	6.59±1.33	6.75±1.16	6.64±0.79	0.915	0.00 [0.00, 0.01]
P1_FHP_	R	6.68±0.74	6.70±0.61	6.57±0.61	6.43±0.45	0.053	0.03 [0.00, 0.07]
	L	6.74±0.75	6.69±0.82	6.60±0.74	6.41±0.58	0.074	0.02 [0.00, 0.07]
P2_OP_	R	4.59±0.66	3.91±0.67	3.78±0.80	3.20±0.83	<0.001*	0.30 [0.19, 0.39]
	L	4.34±0.85	4.14±0.92	3.92±0.84	3.34±0.99	<0.001*	0.14 [0.06, 0.23]
P3_MDBP_	R	4.53±0.72	3.99±0.69	3.87±0.68	3.37±0.75	<0.001*	0.25 [0.14, 0.34]
	L	4.37±0.70	4.29±0.78	3.94±0.65	3.52±0.87	<0.001*	0.16 [0.07, 0.25]

^*^Statistically significant *p* value, *η*^*2*^ eta squared, *CI* confidence interval, *SD* standard deviation,* n* sample size,* R* right, *L* left, *FV* facial vein, *H1*_*MP*_ mental protuberance, *H2*_*MA*_ mandibular angle, *H3*_*APMDB*_ alveolar process of mandible, *H4*_*APMX*_ alveolar process of maxilla, *H5*_*ANS*_ anterior nasal spine, *H6*_*ZA*_ zygomatic arch, *H7*_*R*_ rhinion, *H8*_*N*_ nasion, *V1*_*MOR*_ medial orbital rim, *V2*_*LOR*_ lateral orbital rim, *P1*_*FHP*_ Frankfurt horizontal plane, *P2*_*OP*_ occlusal plane, *P3*_*MDBP*_ mandibular plane

**Fig. 16:**
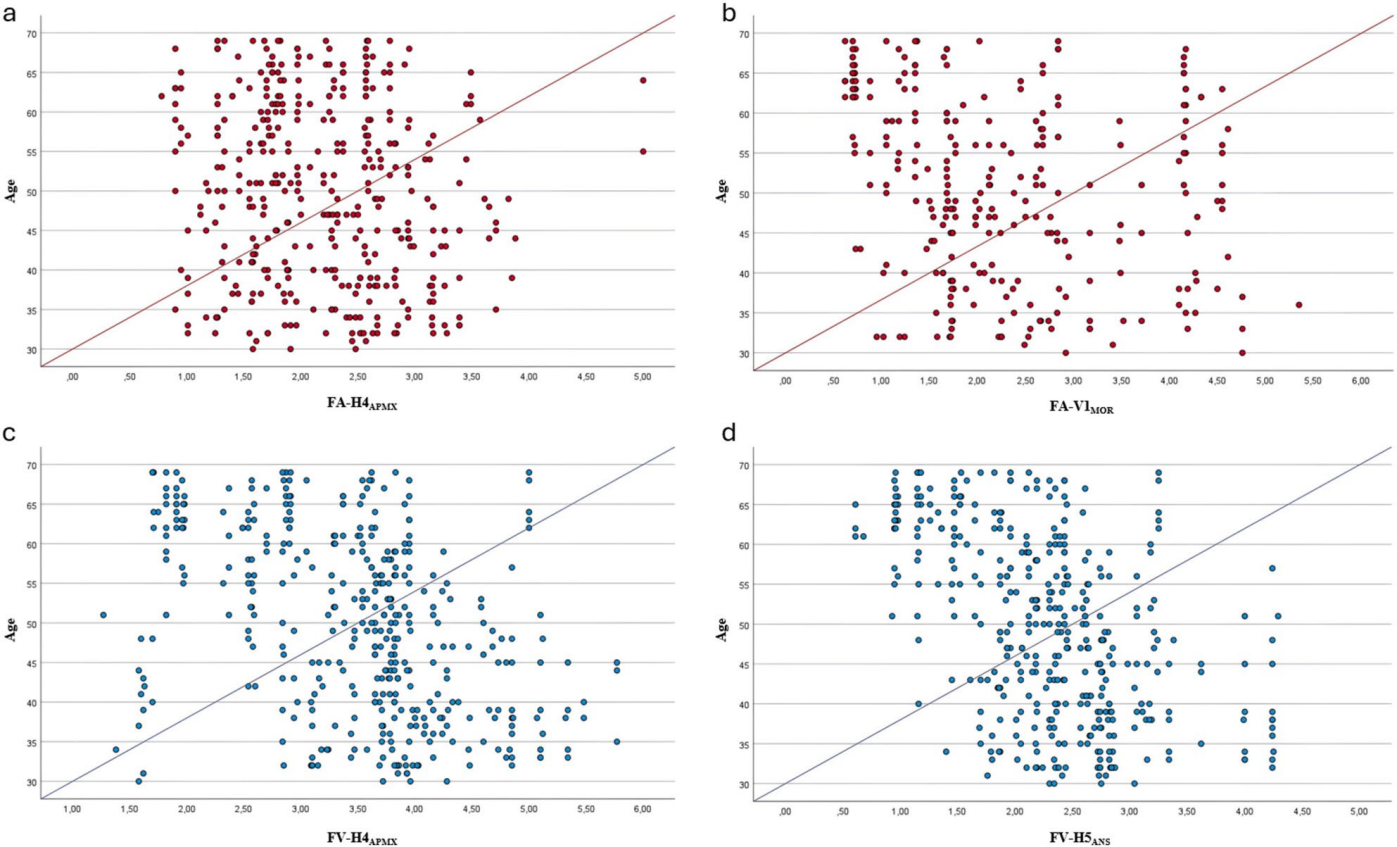
Correlation between age (y-axis) and morphometric distances from anatomical landmarks to facial vessels (x-axis). Correlation between age and the distance from the FA to the alveolar process of the maxilla (H4_APMX_) (**a**) and to the medial orbital rim (FA-V1_MOR_) (**b**). Correlation between age and the distance from the FV to the alveolar process of the maxilla (H4_APMX_) (**c**) and to the anterior nasal spine (H5_ANS_) (**d**)

Figure 16 shows the correlation between age and FA/FV distances to key filler landmarks (H4_APMX_, H5_ANS_, V1_MOR_). In the analyses, significant and strong negative correlations were detected between age and FA-H4_APMX_ (*p* = 0.020, *r* = − 0.168), FA-V1_MOR_ (*p *= 0.002, *r *= − 0.251), FV-H4_APMX_ (*p* = 0.000, *r *=  − 0.556), and FV-H5_ANS_ (*p* = 0.000, *r* = − 0.521).

## Discussion

Although cadaver-based FA and FV studies provide dynamic anatomical data, they are often limited by small sample sizes. In contrast, imaging modalities such as CTA are preferred due to their minimally invasive nature, cost-effectiveness, and high accuracy and resolution in evaluating small-caliber vessels, allowing the assessment of larger sample sizes [3, 10, 11]. However, as a static imaging technique, CTA primarily reflects the topographic route rather than dynamic vascular behavior or precise fascial depth. One of the study’s strengths is the simultaneous evaluation of a large number of FA and FV (*n *= 400) within the same sample group; to our knowledge, this represents the largest sample in which the vascular structures of the face have been assessed using CTA. Most studies assess FA and FV distances using soft tissue landmarks; however, supine CTA may introduce gravity-related soft tissue displacement. Therefore, fixed bony landmarks were used to establish a reproducible skeletal–vascular reference, as the vessel–bone relationship provides a stable anatomical framework despite variations in fascial depth.

Many studies have examined the FA anatomy, but no widely accepted classification exists due to its variable branching patterns. Differences arise from inconsistent naming and inclusion of rare variants. There is especially no consensus on whether the lateral nasal or medial canthal artery is the FA’s terminal branch [2, 3, 12, 13]. Previous cadaver and CTA studies on Asian and Caucasian populations reported FA types as follows: Type I in 0–2%; Type II in 0–5.5%; Type III in 10%; Type IV in 6.6–34.2%; Type V in 39.6%; Type VI in 0–3.3%; Type VII in 44–48%; Type VIII in 20–36.3%; Type IX in 2.3% [2, 4–9]. Studies show the lateral nasal artery is more dominant in Asians, while Caucasian findings vary, with some reporting the medial canthal artery and others the lateral nasal artery as most common [2, 4, 8, 9]. Similarly, our study on a Caucasian population also found the lateral nasal artery (Type VII) to be the most frequent terminal branch (Fig. 14).

Common clinical intervention sites concerning the FA include the glabella, infraorbital region, nose, NLF, lips, and chin [2, 3, 14]. In our study, Type VIII was identified as a high-risk pattern in the glabellar region, with the FA measured at 0.63 cm on the right and 0.67 cm on the left from H8_N_; however, due to other vessels such as the supratrochlear and supraorbital arteries, caution is required throughout the entire glabellar area. Types VI, VII, and VIII were determined to be the most critical for nasal and infraorbital filler procedures. The lower vascular density zones for individuals with Type VI and Type VII FA were defined as follows: for Type VI, regions beyond 1.59 cm on the right and 1.58 cm on the left from H5_ANS_; for Type VII, regions beyond 1.64 cm on the right and 1.55 cm on the left from H7_R_ (Table 2). Gombolevskiy et al. [15] noted that FA type variations raise the risk of vascular complications, especially in the nasal region. Our topographic mapping of lower vascular density zones aims to enhance clinical awareness in these high-risk areas. In the infraorbital region, where both FA and the infraorbital artery are present, meticulous anatomical awareness is essential; lateral approaches may be considered to mitigate risks [1, 10].

NLF interventions are among the most frequent facial procedures [16]. In our study, the vascular patterns most relevant to this area were identified as Type V, Type VI, Type VII, and Type VIII. Among these types, particular attention should be paid to Type V, as it passes through H4_APMX_ and terminates before reaching H5_ANS_. In this FA type, the FA courses 2.33 cm on the right and 1.93 cm on the left of H4_APMX_ (Table 2). The FA–NLF relationship is clinically significant for both aesthetic optimization and vascular risk mitigation. The reported positions of FA relative to NLF in the literature are as follows: 42.9-72.3% medial type, 12.3-23.2% lateral type, 5.7-19.6% cross laterally type, and 7.3-14.3% cross medially type [2, 3, 17]. In our study, consistent with previous reports, the medial type was more common (Fig. 14), and significant associations with multiple morphometric parameters were observed. To enhance awareness during procedures involving the medial NLF, we defined the lower vascular density zones as follows: In the superior region, based on the H5_ANS_ reference, areas closer than 1.53 cm on the right and 1.46 cm on the left; in the inferior region, based on the H4_APMX_ reference, areas closer than 2.23 cm on the right and 2.07 cm on the left (Table 3). While earlier literature reported infrequent vascular complications in the mental region, recent clinical evidence emphasizes the importance of anatomical awareness due to the risk of iatrogenic vascular injury during aesthetic procedures [18]. Our study highlights that Type I FA requires caution during clinical procedures in the chin region, with potential vascular proximity within 4.50 cm (right) and 4.87 cm (left) from H1_MP_, and 4.45 cm (right) and 4.48 cm (left) from H2_MA_ (Table 2). These measurements refer exclusively to the FA and its branches, and it should be considered that other vascular structures in the chin region, such as the mental and submental arteries, may also pose a risk.

The FV is generally more consistent than the FA, so fewer studies focus on it. FV variations are reported between 0.3% and 2% range [6, 7, 19], but Wang et al. [10] found a higher 7% Type II variation in Asians, likely due to ethnic differences compared to mainly Caucasian-focused studies. In our study, Type II and newly defined Type III variations each occurred in 3% of cases (12 out of 400), matching rates reported in Caucasian populations and supporting existing literature. Type II and the newly defined Type III FV variations were equally common (*n *= 6) (Fig. 14). Type I is associated with lower clinical risk due to its deep location, whereas Type III is more critical as it’s superficial and connects to retromandibular and superficial temporal veins. In Type III variation, improper clinical maneuvers may cause to venous complications such as hematoma or obstruction via the superficial temporal vein; however, despite venous–arterial anastomoses, vision loss remains predominantly an arterial complication and is considered a theoretical rather than documented venous risk. Although our study details FV anatomy and variations, FA mapping remains clinically paramount due to the risk of retrograde embolization and the FA’s greater anatomical variability compared with the more consistent FV course.

Male interest in cosmetic procedures has grown significantly in recent years, emphasizing the need to consider anatomical and aesthetic differences between sexes [20]. Studies show that interventions in males should be applied more inferomedially to enhance masculinity and reduce vascular risks due to the posterior location of vessels [21]. Common sites for clinical interventions include cheeks and tear troughs, where anatomical awareness near the orbital rim recommended to avoid major infraorbital vessels [20]. Our findings identify the intersection of H5_ANS_ (horizontal plane) with V1_MOR_ and V2_LOR_ (vertical plane) as the most dangerous area for infraorbital region.

Aging-related bone resorption and soft tissue loss, especially in the maxilla, alters facial contours-retracting the infraorbital rim, deepening the tear trough, flattening the zygomatic arc, widening the piriform aperture, and accentuating the NLF [22]. Gelezhe et al. [23] showed that aging reduces the NLF–FA distance. Similarly, our study found that FA and FV lie closer to bone landmarks in the aging maxilla and mandible (Tables 6 and 7). Specifically, due to maxillary resorption, the retraction of the infraorbital rim inward and backward, along with the prominence of the tear trough, led to a significant decrease in the FA-V1_MOR_ distance. Similar bone volume losses in the mandible manifested as age-related distance reductions in measurements such as FA-H1_MP_ and FV-H1_MP_. These findings indicate that age-related bone resorption may approximate vessels to bony landmarks, increasing the risk of vascular injury, especially during deep supraperiosteal procedures.

Our study has four main limitations. First, it is a single-center study conducted only on individuals from the Turkish population. Second, the lack of similar studies examining the relationship between FA and FV and bony landmarks has made directly comparing our findings difficult. Third, although our sample was larger than previous studies, multi-center research with diverse ethnic groups is needed to clarify the effects of ethnicity and comorbidities and improve generalizability. Fourth, and most importantly, all distances represent statistical averages; given the high variability of facial vasculature, these defined topographic zones do not provide absolute protection but serve as adjunctive guidance for clinical navigation. They should never replace standard clinical risk-management protocols—such as aspiration, slow delivery, or ultrasound guidance—or individualized clinical judgment.

## Conclusion

Understanding FA and FV anatomy is key to identifying topographic patterns of vascular distribution and mitigating clinical risks during various facial procedures. In this study, we identified novel morphological types of FA and FV, with the lateral nasal variant being the most frequent FA pattern, typically coursing medial to the NLF. Our findings demonstrate that distances between vessels and bony landmarks are significantly influenced by gender and age-related bone remodeling, being greater in males and younger individuals. This large-scale study uniquely evaluates both the FA and FV, providing a comprehensive anatomical framework that helps clinicians mentally visualize the most common vessel locations relative to stable reference landmarks, thereby enhancing procedural safety and anatomical awareness.
